# Changing Epidemiology of Myocarditis in Australia: A Population-Based Cohort Study

**DOI:** 10.3390/jcm13237111

**Published:** 2024-11-24

**Authors:** Timothy Nathan Kwan, Gemma Kwan, David Brieger, Leonard Kritharides, Vincent Chow, Austin Chin Chwan Ng

**Affiliations:** 1 Department of Cardiology, Concord Hospital, The University of Sydney, 1 Hospital Road, Concord, Sydney, NSW 2139, Australia; 2 Department of Immunology, Royal Prince Alfred Hospital, The University of Sydney, 50 Missenden Rd., Camperdown, Sydney, NSW 2050, Australia

**Keywords:** myocarditis, heart failure, epidemiology, COVID-19, Australia

## Abstract

**Background**: Myocarditis is a serious disease that has drawn increasing attention due to its association with COVID-19 and vaccination. This study investigates the epidemiology of myocarditis beyond the COVID-19 pandemic, including its incidence and outcomes over time. **Methods**: We analyzed the population-wide retrospective data from the Admitted-Patient-Data-Collection database of patients admitted to hospitals in New South Wales (NSW), Australia, with a diagnosis of myocarditis from 2001 to 2022. The incidence of myocarditis, changing classification of myocarditis over time, and complications of myocarditis over time were all calculated. **Results**: There were 4071 patients diagnosed with their first episode of myocarditis, with a median age of 42 years old, and 66% were male. The incidence of myocarditis in NSW has tripled over 20-years to 8.3 per-100,000-persons by 2022. Reactive myocarditis (i.e., myocarditis within 30-days of a respiratory or digestive illness) accounted for 38% of first presentations of myocarditis. Post COVID-19 myocarditis, a subset of reactive myocarditis, accounted for 42% of myocarditis admissions since the onset of the COVID-19 pandemic in Australia. Eight percent of patients had a background history of malignancy, and 6% had a history of autoimmune disease. In-hospital mortality was 4.5% during the entire study period but has been falling by 11% per year. During follow up, most readmissions for myocarditis occurred within 6-months; with 5.1% recurrence at 6-months compared to only 6.7% at 5-years. **Conclusions**: Myocarditis is an important condition with increasing incidence in Australia and with markedly changing characteristics in the pandemic and post pandemic era.

## 1. Introduction

Myocarditis is defined as myocardial inflammation and is an important contributor to the global burden of cardiovascular disease with both acute and chronic sequelae [1]. However, it is a heterogeneous disease that is difficult to precisely define and characterize [2]. The disease can present with chest pain, heart failure, or arrhythmias but may even be asymptomatic [3,4]. Retrospective studies describe 30–50% in hospital mortality for myocarditis patients requiring extracorporeal membrane oxygenation [5]. By contrast, studies that include a broader spectrum of myocarditis identify a 1-year mortality of about 8%, only 4-fold higher than a reference population [6].

Myocarditis has many possible classifications, one of which is to categorize by cause: reactive post-infection, toxin-induced, particularly from medications, or as a sequelae of an autoimmune syndrome [7]. Myocarditis that is reactive to viral infections or secondary to viral persistence in the myocardium is generally considered the most common cause of myocarditis [8]. Many publications have recognized the rising incidence of myocarditis because of COVID-19 [9,10], however, there is a scarcity of epidemiological research describing the broader landscape of myocarditis according to etiology.

Variable reports from global studies have illustrated the changing epidemiology of myocarditis. Data from the Global Burden of Disease database demonstrated that although the absolute number of myocarditis cases has been increasing, there is a small but significant reduction in the age-adjusted incidence of myocarditis up to 2019 [11,12]. By contrast, the incidence of myocarditis in the United States (US) steadily increased more than 50% from 95 to 144 per-million between 2005 and 2014 [13]. In keeping with this observation, a rise in mechanical circulatory support is observed for treating myocarditis [13]. Many smaller studies have reported much higher incidence of myocarditis after COVID-19 [14]. One American study demonstrated that COVID-19 patients had a risk of myocarditis 16 times higher than all other patients [15]. COVID-19 vaccination has also been related to myocarditis both epidemiologically and mechanistically [16,17].

Given the clinical importance of the condition, variability in reported epidemiology of myocarditis to date, and lack of post pandemic population-wide unselected long-term studies of myocarditis in Australia or elsewhere, we performed a population-wide linkage study to examine the epidemiology of all myocarditis admissions in the most populous state of Australia, New South Wales (NSW), between 2001 and 2022.

## 2. Materials and Methods

In this population-wide cohort study, data were acquired through the Centre for Health Record Linkage (CHeReL). Data were linked from the NSW Admitted Patient Data Collection (APDC) database and the NSW Registry of Births, Deaths and Marriages. The APDC contains more than 97% of NSW healthcare facility admission administrative records. It contains standard demographic data and all diagnoses and procedures from each hospital admission [18]. A unique Project Personnel Number (PPN) for each individual patient allows capture of recurrent admission events during the study period and linkage to the death registry. Admissions dated between 2001 and 2022 were included.

Patients were included in the present study if they had a diagnosis of myocarditis recorded according to the International Statistical Classification of Diseases and Related Health Problems, Tenth Revision, Australian Modification (ICD-10AM) codes, either as a principal or secondary diagnosis during a hospital admission, while data on procedures were based on the Australian Classification of Health Interventions codes (Table S1). The NSW population-structure data were obtained from the Australian Bureau of Statistics website [19].

**Figure 1:**
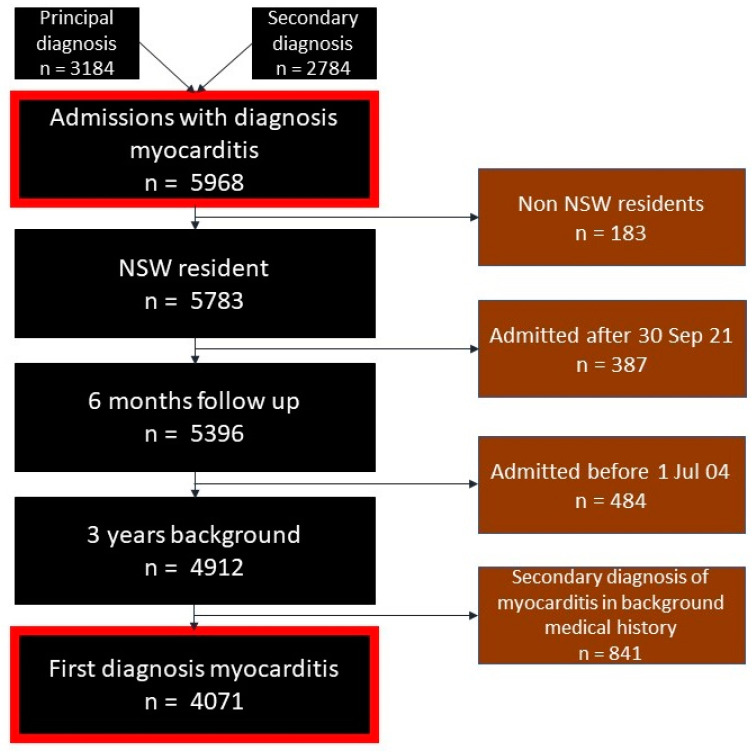
Inclusion and exclusion criteria.

Two cohorts were analyzed in the present study (Figure 1). Firstly, for the purpose of reporting the incidence of myocarditis (total admission caseloads), recurrent admissions for myocarditis were included. Secondly, for all calculations beyond reporting the admission caseloads, admissions for recurrent myocarditis were excluded, and only admissions for the first episode of myocarditis were analyzed to determine mortality and other complication rates post-myocarditis. In this second outcome study cohort, patients were prespecified in our analytical plan to have a minimum of 6-months of follow-up, 3-years of look-back to identify recurrent myocarditis, and were all NSW residents to reduce loss to follow-up. To accommodate the follow-up requirements, patients were excluded if they were admitted before 1 July 2004 or after 30 September 2021. The period before 2004 was used for background diagnoses, and the period after 2021 was used for complications and mortality. By contrast, for reporting total admission caseloads (the first cohort), all available data were used from 2001 to 2022.

For this study, the following classifications were adopted: autoimmune, malignant, reactive, or COVID-19-related myocarditis, with associated rates counted. Autoimmune and malignant myocarditis were defined as having a background history of these conditions either as a primary or secondary diagnosis from the index or any prior admission. Reactive myocarditis was defined as having an admission for a respiratory or digestive system condition in the 30-days prior to the myocarditis admission. COVID-19 myocarditis was defined as having an admission for COVID-19 in the 30-days prior to the myocarditis admission and considered a subset of reactive myocarditis. Biopsy-proven definitive myocarditis was defined as having a diagnosis of myocarditis during the same admission as an endomyocardial biopsy. This categorical system to classify myocarditis in an administrative database was developed for this specific study.

We reported summary statistics at myocarditis admission, which included the socioeconomic index for area (SEIFA) based on their Statistical Area Level 2 (SA2) and the Australian Statistical Geography Standard (ASGR) [19,20]. To calculate the incidence of myocarditis, we used a standard method to adjust for NSW population size stratified by age groups and sex per-year to derive the annual incidence rate of myocarditis admissions per-100,000-persons [21]. Where data from the full year were not available (for the first and last year of follow up), data were linearly extrapolated to the full year. Background conditions were attained from both records from the index admission as well as any prior admissions during the lookback period to 2001. Significance was set at *p* < 0.05.

The risk of myocarditis complications, including heart failure, ventricular arrhythmia, syncope, myocarditis readmission, defibrillator insertion, and heart transplant, was calculated using the Fine-and-Gray method, competing against death. The significance of changes in features of myocarditis was quantified with logistic regression for binary variables and with linear regression for continuous variables on the individual data rather than binned data. Multivariable analysis was performed, adjusting for age, sex, and Charlson comorbidity index (CCI). The patterns of demographics, outcomes, and complications were plotted over time, dividing the time of index admission into quintiles and deciles.

The study protocol conforms to the ethical guidelines of the 2013 Declaration of Helsinki. Ethics approval was granted by the NSW Population and Health Services Research Ethics Committee, reference number: 2013/09/479, which also granted a waiver of the usual requirement for the consent of the individual to the use of their health information. All patient data were deidentified and analyzed anonymously.

## 3. Results

There were 4071 patients diagnosed with their first episode of myocarditis between 1 July 2004 and 30 September 2021 (Figure 1). The median age was 42 years old (interquartile range [IQR] 27–59 years old), and two-thirds were male (Table 1). They were mostly (92%) admitted to public hospitals and were mostly (89%) unplanned presentations requiring emergency treatment within 24 h.

**Figure 2:**
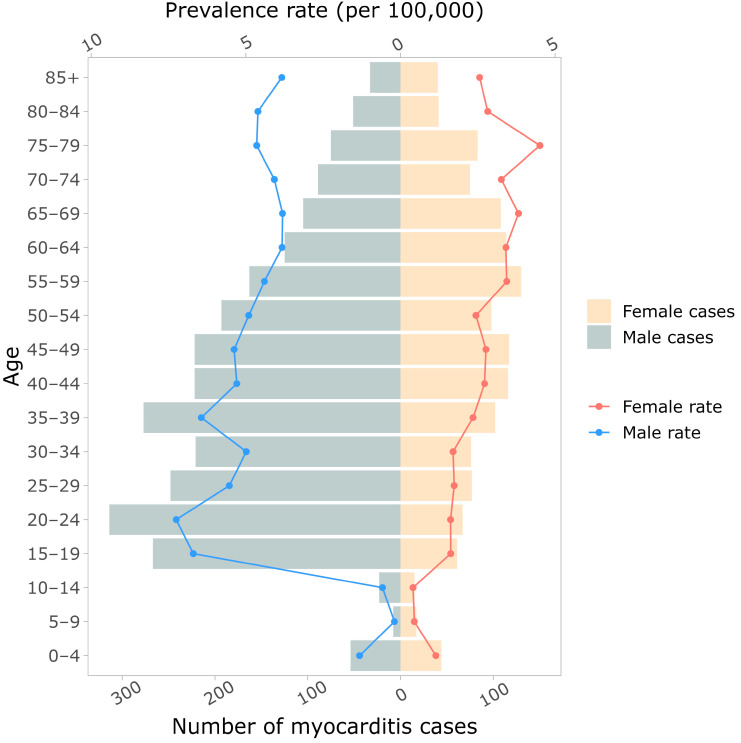
Population demographics of first presentation of myocarditis from 2004 to 2021 separated by age and sex.

The frequency of myocarditis was highest in young and middle-aged adults (15–60 years old), and in this group, more males than females were affected. At the extremes of age, myocarditis rate did not vary based on sex (Figure 2). This demographic appearance of myocarditis has remained stable over the past 20 years (Figure S1).

Patients with myocarditis were mostly (74%) living in major cities (Table 1). The remoteness of patients presenting with myocarditis was distributed similarly to the NSW general population (chi-squared test *p* = 0.22). Patients’ socioeconomic index mean SEIFA score was 1000 (median 996, IQR 939–1065), almost identical to the reference value for Australia, which is set to 1000.

Patients admitted with their first episode of myocarditis were moderately comorbid with a median CCI of 1 (Table 1). One-quarter had been previously diagnosed with heart failure, and over one-quarter had ischemic heart disease (Table 1).

**Figure 3:**
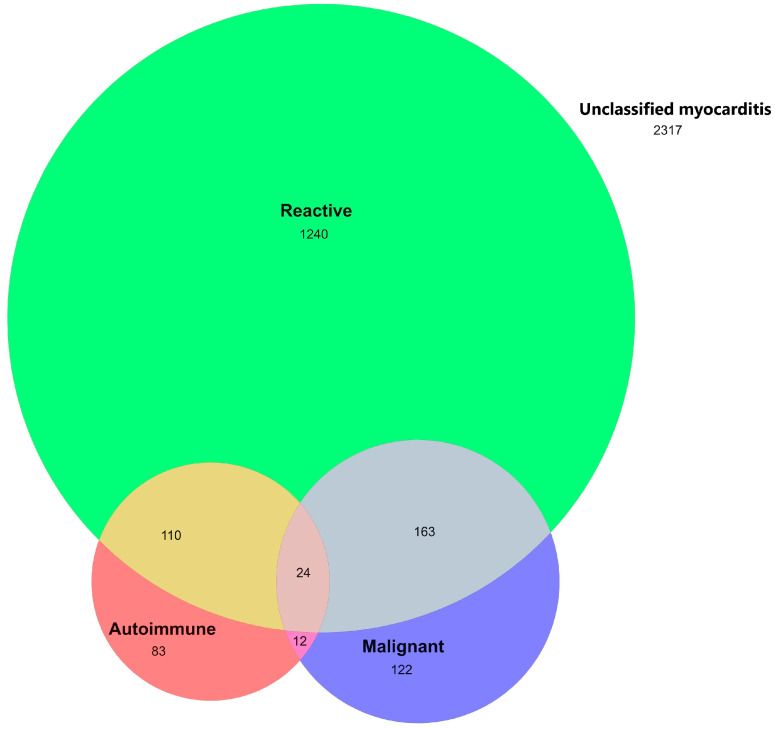
Frequency of myocarditis according to possible classification. Autoimmune, malignant etiologies were defined by having a background of autoimmune disease, or, respectively. Reactive myocarditis was defined as having a history of a respiratory or digestive system infection in the past 30 days.

Autoimmune myocarditis accounted for 6% of patients with myocarditis, and malignant myocarditis accounted for 8%. Patients with reactive myocarditis accounted for 38% of cases (Figure 3). Eighteen percent of reactive myocarditis (7% of all myocarditis) was associated with COVID-19. However, since the onset of the pandemic in Australia on 25 January 2020, 281 (42%) myocarditis cases have been within 30-days of an admission with COVID-19. In this post pandemic era, a larger proportion (50%) of all myocarditis has been reactive (Figure S2). Myocarditis with concomitant IHD accounted for 18% of myocarditis. Fifty-seven percent of patients had idiopathic myocarditis, which was defined as myocarditis without autoimmune, malignant, or reactive myocarditis. Females were three times as likely to have an autoimmune classification for their myocarditis than men, and females also had slightly higher rates of myocarditis that could be classified as malignant or reactive (Table S2).

Biopsy rates during or after myocarditis diagnosis were not significantly more common in major cities compared to rural areas (Table S3). Patients in rural areas were similarly unwell as measured by in hospital mortality, ICU admission rates, and rates of mechanical ventilation.

### 3.1. Caseload of Myocarditis Admissions and Temporal Trends in Characteristics During Study Period

**Figure 4:**
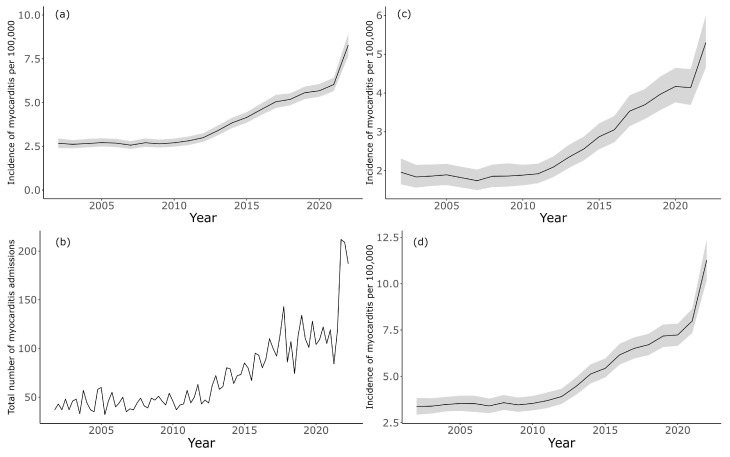
Age and sex standardized incidence of all myocarditis admissions over time: (**a**) all patients, (**b**) unadjusted incidence for all patients, (**c**) female patients, (**d**) male patients. Shaded area indicates 95% credible intervals.

There were 5968 unique admissions for myocarditis. The age and sex-standardized incidence of myocarditis admissions remained relatively static at about 2.5 cases per-100,000 until 2012 after which there was a gradual rise (Figure 4a). There was an inflexion point in 2021 during which the growth rate in the incidence of myocarditis accelerated. By 2022, the incidence of myocarditis was about 8 cases per-100,000 with no sign of slowing. This trend was similar irrespective of age range and trended the same as the crude incidence data (Figure 4b). The trend was similar for both females and males, although the incidence of myocarditis was higher in males (Figure 4b,c).

There was no significant change in sex ratio during the study’s 20-year period (*p* = 0.209, Figure S3a). There was a significant, albeit small, absolute increase in the average age of myocarditis patients by 2.2 months per-annum, accounted for entirely by the past 10-years (*p* = 0.002, Figure S3b). In addition, the overall comorbidity burden (based on CCI) of admitted patients with myocarditis has increased over time (*p* < 0.001, Figure S3c).

The proportion of myocarditis associated with autoimmunity has remained relatively static (*p* = 0.563, Figure S3d) although the proportion associated with malignancy has increased (*p* < 0.001, Figure S3e). The proportion of reactive myocarditis has increased and appears fully attributable to the onset of the COVID pandemic (*p* < 0.001, Figure S3f): the proportion of reactive myocarditis excluding COVID-19 was static over time (*p* = 0.227, Figure S3g). The proportion of myocarditis patients with concomitant IHD marginally increased during the study period (*p* = 0.046, Figure S3i). Biopsy proven definitive myocarditis has been stable (Figure S4), but in the context of rising rates of myocarditis in general, there has been a reduction in the proportion of myocarditis patients being biopsied during their index admission (*p* = 0.031, Figure S3j).

### 3.2. Outcomes After Myocarditis Diagnosis

**Figure 5:**
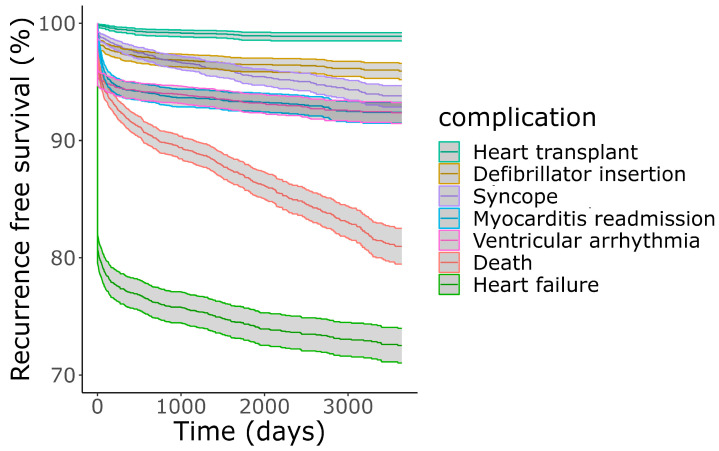
Incidence of complications of myocarditis over time according to competing risk analysis. Complications such as heart failure, which sometimes occurred during the index admission for myocarditis, were regarded as instantaneous. The complications from least to most frequent at end of follow up were heart transplant, defibrillator insertion, syncope, myocarditis readmission, ventricular arrhythmia, death, and heart failure.

Total inpatient mortality was 4.5% (182/4071, Table 1). The 30-day mortality was similar at 4.3% (172/4071), and the 5-year mortality was 13.3% (Figure 5). Patients with myocarditis stayed in hospital a median of 4 days (Table 1). Fifteen percent required ICU admission, half of which (8%) required mechanical ventilation. Patients admitted to ICU were hospitalized for a median of 12 days.

Readmissions for myocarditis were relatively uncommon, and the vast majority occurred within 6-months of initial presentation for myocarditis: 5.1% were readmitted for myocarditis within 6-months and 6.7% by 5-years (Figure 5). Complications of myocarditis were most common during the index admission but continued to rise steadily over years after the index presentation. The most common morbidity of myocarditis as either a presenting complaint or complication during index admission was heart failure (19%) (Table 2). This was also a common delayed complication, with an additional 7% admitted for heart failure within 5-years of myocarditis presentation. Ventricular arrhythmia was common during index admission (4.6%). Defibrillators were inserted in 1.6% of patients during their index admission for myocarditis. Among those who did not have a defibrillator inserted during index admission, 1.9% had one inserted over the subsequent 5-years. Heart transplants were uncommon at 0.9% by 5-years (Figure 5), and only 16% of these were performed during the index admission for myocarditis.

In-hospital mortality following myocarditis admissions fell over the past 20-years from a crude 9.6% from 2001 to 2005 to a crude 3.4% from 2018 to 2022. Using logistic regression, there was an adjusted 11% reduction in in-hospital mortality per-year for the past 20-years (Figure S3k). The proportion of myocarditis patients admitted to the ICU and requiring mechanical ventilation fell slightly by 2% and 4%, respectively (Figures S3l and S3m). There has been a slight reduction in heart failure complicating myocarditis over the past 10-years following a peak in the 2009–2013 year-group (Figure S3n). The chances of ventricular arrhythmia or syncope have not significantly changed (Figures S3o,p), however, there have progressively been more defibrillators inserted for myocarditis (Figure S3q). Heart transplantation remains a rare intervention during an admission for myocarditis throughout the study period (Figure S3r).

## 4. Discussion

The present study examined the epidemiology of myocarditis and showed the frequency of myocarditis diagnosis has been rising steeply, particularly in the past 5–10 years. The proportion of biopsy-proven myocarditis has been falling, while the incidence of myocarditis associated with malignancy has increased. Since 2020, COVID-19 myocarditis has accounted for approximately half of myocarditis admissions, however, other reactive myocarditis has persisted at a steady rate. Major complications of myocarditis are death, heart failure, myocarditis readmission, and arrhythmia; all of which are common. Although patients with myocarditis have been becoming more comorbid with a higher CCI, complications, including death and ICU admission, are less frequent.

### 4.1. Relationship with Existing Literature

The preponderance of male patients diagnosed with myocarditis and the absolute decline in cases with age have been reported in previous research [22]. This study confirms that young males are a group disproportionately affected by myocarditis [23]. In the present study, this was not related to any classical risk factors for myocarditis, as autoimmune disease, malignancy, and even reactive myocarditis were all higher proportionately in female patients than male patients. Similarly, although mechanisms have been purported in the literature, the mechanism for this gender disparity remains unknown [24]. Triggers for COVID-19, such as COVID-19 vaccination, have not been shown to differentially affect males compared to females [25].

Longitudinal data is less well reported than cross sectional data, and the largest international studies (last in 2019) demonstrated age-adjusted myocarditis incidence has been stable or slightly shrinking [11,12]. Smaller studies, outside of Australia, have shown clinically diagnosed myocarditis rates are rising [13], especially post COVID-19 [10,14,15]. It was realized early in the pandemic, first through case reports, that myocarditis commonly complicated COVID-19 [26]. More recently, it has also been noted that COVID-19 myocarditis can incur a higher mortality rate than myocarditis in general [27,28]. In the United States there was a rise of inpatient myocarditis encounters in 2020 by 42% compared to 2019 [15]. The anecdotal experience of a large healthcare system in Washington DC found that although the rise in myocarditis cases in 2020 was 40% higher than the 3 pre-pandemic years, by 2022 the in-hospital crude myocarditis incidence was 105% higher [29]. In France, myocarditis diagnoses were found to be 11% higher in 2020 compared to the three prior years [30]. Surprisingly, in Italy a small reduction in post-pandemic incidence of myocarditis was reported [31].

Data from the Global Burden of Disease database was used to demonstrate Oceania has an age-standardized myocarditis incidence rate of about 16 per-100,000 both in 1990 and 2019, which was also the global average in 2019 [12]. The incidence rate is about half this in the present study. It is, however, close to the incidence rate of 1.15–14 per 100,000 shown in a large Polish registry and similarly demonstrates the preponderance of myocarditis in young males [32,33]. As far as we are aware, more granular data describing the epidemiology of myocarditis comparable to our study is lacking [12]. Differences in study design and Oceania countries included in the GBD database likely account for the finding’s disparity. The in-hospital mortality rate from acute myocarditis of 4.5% was on the upper end of the mortality rate reported in other epidemiological studies of 1–7% [34,35]. However, given myocarditis mortality was found to be declining in the present study, the in-hospital mortality of 3% from 2018 to 2022 is consistent with similar recent literature. Another possible explanation for the slightly higher death rate seen in the study was that Oceania has previously been found to have slightly higher death rates from myocarditis than other countries [36].

### 4.2. Implications of Study Findings

The current study underlines the rising incidence of myocarditis, especially post-pandemic. This study does not elucidate the mechanism by which COVID-19 leads to myocarditis, but clearly many clinicians are recognizing myocarditis within 30-days of COVID-19 infection, suggesting this may be a period to be particularly vigilant for myocarditis. Another growth area of myocarditis incidence is in patients with malignancy, possibly due to the increasing use of cardiotoxic chemotherapy and immune checkpoint inhibitors. Clinicians should be vigilant for myocarditis in this at-risk population. Surprisingly, a large proportion of myocarditis cases (18%) are diagnosed in the context of concomitant IHD, despite coronary artery disease being considered an exclusion criterion for the diagnosis of myocarditis [3]. Whilst being aware of possible myocarditis in this ischemic population, it is also important to be wary of misdiagnosis.

The nature of myocarditis is changing, as although myocarditis patients are becoming more comorbid in Australia, the mortality after myocarditis diagnosis is decreasing. One likely explanation is that myocarditis is being diagnosed even when its manifestations are subtle due to more sensitive non-invasive techniques becoming available, including high-sensitivity troponin assay and cardiac magnetic resonance imaging (MRI) [37]. Cardiac MRI is increasingly available [38] and in this context, syndromes such as myocardial infarction with no obstructive coronary arteries are being newly classified as myocarditis [39]. Other outcomes, such as ICU admission and heart failure, are becoming less common, although this trend is only evident after adjusting for comorbidities. The frequency of ventricular arrhythmia and syncope is not changing, however, invasive strategies to manage complications of myocarditis, including defibrillator insertion and even heart transplantation, are rising.

There are immediate and long-term complications of myocarditis, including heart failure and ventricular arrhythmia, that are important to monitor. Our study demonstrates that most complications occur early, and most recurrences of myocarditis are within 6-months of the index presentation. Similarly, implantable cardioverter defibrillators, which may represent treatments of ventricular arrhythmia or primary prophylaxis for severely impaired left ventricular ejection fraction, were mostly inserted during the index admission. Nevertheless, clinicians should maintain vigilance in monitoring post-myocarditis patients, as close to 1 in 50 patients subsequently required defibrillator insertions over 5-years follow-up.

### 4.3. Strengths and Weaknesses

The present study reports contemporary longitudinal data on the incidence of myocarditis in an unselected population-wide cohort study in the largest state of Australia. Our large cohort allows for stratification and analysis of subgroups. However, our study raises several etiological questions that cannot be answered due to its observational nature, including the mechanism by which COVID-19, malignancy, and other conditions are associated with myocarditis. This study also cannot demarcate what social or health changes have caused the rapid rise in myocarditis incidence over the past 20-years, although factors such as high-sensitivity cardiac troponin assays and cardiac MRI may be proposed as hypotheses. Secondly, relevant details are missing, such as medications and laboratory results. One important implication of this is that the categorization of myocarditis into malignant, autoimmune, or reactive was inferred from a patient’s previous diagnoses rather than confirmed by specific investigations. However, even the presence of investigations such as viral serology are mere associations and cannot confirm etiology [3]. Thirdly, it could not be proven whether myocarditis or its complications were acute or chronic diagnoses, as these data were not available. The three year look back strategy did exclude previously diagnosed myocarditis, which provided some evidence that included cases of myocarditis were the index diagnoses.

Finally, the diagnosis of myocarditis is challenging given the low rate of endomyocardial biopsies and lack of pathology results. As this and other studies have shown, myocarditis is increasingly being diagnosed non-invasively [40]. Although publications are being released to guide the non-invasive diagnosis of myocarditis, this can only result in clinically suspected myocarditis rather than a definitive diagnosis of myocarditis [3]. It is likely there were many false positives and false negatives in myocarditis diagnosis at a population-level. Differences between hospitals and individual clinicians in the robustness of the workup of myocarditis could not be elucidated by this study. Nevertheless, similar administrative data performed in Sweden found excellent (96%) positive predictive value for myocarditis [41]. Furthermore, the classification of myocarditis etiology is difficult, especially with administrative data. In the present study, a background of autoimmune disease and malignancy was used to classify myocarditis, although in many cases these background diagnoses would have been incidental rather than causative. Similarly, reactive myocarditis was defined by the occurrence of an ostensibly causative infection or trigger in the past 30 days, although it is impossible to verify whether this was causative. Studies validating the classification of myocarditis in administrative databases are needed. The threshold of 30 days was based on biological plausibility and because this tends to be the duration required for symptoms to develop after a viral exposure [34,42]. A similar approach to classifying COVID-19 related myocarditis has recently been adopted using the same 30-day threshold [27].

## 5. Conclusions

Myocarditis is an important condition associated with high morbidity and mortality. The rate of myocarditis has been rising in Australia over the past 10-years, especially since the onset of the COVID-19 pandemic. The study highlights the changing landscape of myocarditis in terms of associated comorbidity and complications and aims to propel further studies in this field.

## Figures and Tables

**Table 1:** Summary features of patients admitted for myocarditis.

Feature	Value
Basic features	
Male	66.1% (2690/4071)
Age	42.4 (27.2–58.7)
Emergency	88.5% (3279/3705)
Private hospital	8.2% (333/4071)
Private insurance	27.6% (1125/4071)
Major city ^a^	74.2% (3007/4055)
Rural ^b^	5.8% (235/4055)
SEIFA ^c^	995.8 (938.6–1065.3)
Male	66.1% (2690/4071)
Admission outcomes	
In hospital mortality	4.5% (182/4071)
Admission duration (days)	4.1 (2–10)
ICU admission	15.4% (625/4071)
ICU duration (hours)	94 (39–182)
Mechanical ventilation	7.7% (315/4071)
Mechanical ventilation in ICU	92 (32.5–197)
In hospital mortality	4.5% (182/4071)
Admission duration (days)	4.1 (2–10)
Male	66.1% (2690/4071)
Background conditions	
Charlson comorbidity index	1 (0–3)
Cardiac disease	
Background congestive cardiac failure	23.5% (955/4071)
Background atrial fibrillation	12.5% (507/4071)
Background ventricular arrhythmia	6.2% (253/4071)
Background pulmonary hypertension	2.1% (86/4071)
Background CKD	4.6% (187/4071)
Background congestive cardiac failure	23.5% (955/4071)
Background atrial fibrillation	12.5% (507/4071)
Background ventricular arrhythmia	6.2% (253/4071)
Cardiovascular risk factors	
Background ischemic heart disease	27.3% (1110/4071)
Background diabetes	10.4% (423/4071)
Background hypertension	26.8% (1090/4071)
Background hyperlipidemia	7.9% (320/4071)
Background smoking	45.3% (1846/4071)
Background emphysema	11.9% (485/4071)
Autoimmune conditions	
Background autoimmunity	5.6% (229/4071)
Background connective tissue disease	3.1% (128/4071)
Background systemic lupus erythematosus	1.2% (50/4071)
Background rheumatoid arthritis	1.1% (45/4071)
Background sarcoidosis	0.8% (32/4071)
Background inflammatory bowel disease	1.5% (61/4071)
Background human immunodeficiency virus	0.4% (18/4071)
Background myositis	1.5% (62/4071)
Background pericarditis	5.6% (227/4071)
Malignancy	
Background malignancy	7.9% (321/4071)

a. Major city defined by Australia Statistical Geography Standard of 1; b. Rural defined by Australian Statistical Geography Standard of 3 or more; c. SEIFA = Socio-Economic Indexes for Areas; Continuous variables reported as median (IQR). Binary variables reported as mean (n). Where denominators were less than 4071, this indicated missing data.

**Table 2:** Incidence of inpatient complications during admission for myocarditis (n = 4071).

Complications	Incidence During Admission for Myocarditis			Cumulative Incidence by 5 Years After Myocarditis		
	Percentage	95% Confidence Interval	Absolute Number	Percentage	95% Confidence Interval	Absolute Number
Death	4.5	3.9–5.2	182	13.3	14.4–12.2	497
Defibrillator insertion	1.6	1.2–2.0	65	3.5	3.0–4.1	136
Heart failure	19.2	18–20.4	780	26.4	25–27.8	1034
Heart transplant	0.2	0.1–0.4	7	1.1	0.8–1.5	43
Syncope	0.9	0.6–1.2	36	4.4	3.8–5.1	160
Ventricular arrhythmia	4.6	4–5.3	187	6.8	6.0–7.6	265
Myocarditis readmission	-	-	-	6.7	5.9–7.5	263

## Data Availability

The original data presented in the study are available in NSW Admitted Patient Data Collection (APDC) database and the NSW Registry of Births, Deaths & Marriages. These data can be attained on application to the NSW government but are not owned by the study authors.

## Supplementary Materials

The following supporting information can be downloaded at: https://www.mdpi.com/article/10.3390/jcm13237111/s1, Table S1: The International Classification of Disease (ICD-10AM) and Australian Classification of Health Intervention (ACHI) codes used in the present study. Table S2: Comparative trends for myocarditis based on sex. Table S3: Comparative trends for myocarditis in major cities and rural areas. Figure S1: Population demographics of first presentation of myocarditis per year separated by age and sex. Figure S2: Frequency of myocarditis according to possible classification since 25 January 2020. Figure S3: Changing myocarditis demographics over time from 2001 to 2022. Figure S4: Incidence of definitive myocarditis admissions in NSW over time plotted with total myocarditis admissions.
