# Supplementary material for: Changing Epidemiology of Myocarditis in Australia: A Population-Based Cohort Study
Source: J Clin Med. 2024 Nov 24;13(23):7111. doi: 10.3390/jcm13237111 (PMC11642529; doi:10.3390/jcm13237111)
Supplement: Supplementary file 1 [file jcm-13-07111-s001.zip › jcm-3281741-supplementary.pdf]

Supplementary data

Table S1: The International Classification of Disease (ICD-10AM) and Australian Classification of Health Intervention (ACHI) codes used in the present study

Diagnosis	ICD-10AM or ACHI code
Atrial fibrillation	I48, I48.0, I48.1, I48.2, I48.3, I48.4, I48.9
AIDS	B20, B21, B22, B23, B23.0, B23.8, B24
Chronic kidney disease	N18, N18.3, N18.4, N18.5, N18.9, N19, I12.0, I13.1, U87.1, Z49, Z49.0, Z49.1, Z49.2, Z94.0, Z99.2
Covid	U06.0, U07.1, U07.2, U07.7
CTD	M30, M30.0, M30.1, M30.2, M30.3, M30.8, M31, M31.0, M31.1, M31.2, M31.3, M31.4, M31.5, M31.6, M31.7, M31.8, M31.9, M32, M32.0, M32.1, M32.8, M32.9, M33, M33.0, M33.1, M33.2. M33.9, M34, M34.0, M34.1, M34.2, M34.8, M34.9, M35, M35.0, M35.1, M35.2, M35.3, M35.4, M35.5, M35.6, M35.7, M35.8, M35.9, M36, M36.0, M36.1, M36.2, M36.3, M36.4, M36.8
Dilated cardiomyopathy	I42.0
Dementia	F00, F00.0, F00.1, F00.2, F00.9, F01, F01.0, F01.2, F01.3, F01.8, F01.9, F02, F02.0, F02.1, F02.2, F02.3, FO2.4, FO2.8, F03, F05.1, U79.1, G30, G30.0, G30.1, G30.8, B30.9
Diabetes	E10, E10.0, E10.00, E10.01, E10.02, E10.1, E10.10, E10.11, E10.12, E10.13, E10.14, E10.15, E10.16, E10.2, E10.20, E10.21, E10.22, E10.23, E10.29, E10.3, E10.30, E10.31, E10.32, E10.33, E10.34, E10.35, E10.36, E10.39, E10.4, E10.40, E10.41, E10.42, E10.43, E10.49, E10.5, E10.50, E10.51, E10.52, E10.53, E10.59, E10.6, E10.60, E10.61, E10.62, E10.63, E10.64, E10.65, E10.69, E10.7, E10.70, E10.71, E10.73, E10.8, E10.80, E10.81, E10.9, E10.90, E10.91, E11, E11.0, E11.00, E11.01, E11.02, E11.1, E11.10, E11.11, E11.12, E11.13, E11.14, E11.15, E11.16, E11.2, E11.20, E11.21, E11.22, E11.23, E11.29, E11.3, E11.30, E11.31, E11.32, E11.33, E11.34, E11.35, E11.36, E11.39, E11.4, E11.40, E11.41, E11.42, E11.43, E11.49, E11.5, E11.50, E11.51, E11.52, E11.53, E11.59, E11.6, E11.60, E11.61, E11.62, E11.63, E11.64, E11.65, E11.69, E11.7, E11.70, E11.71, E11.72, E11.73, E11.8, E11.80, E11.81, E11.9, E11.90, E11.91, E12, E12.0, E12.00, E12.01, E12.1, E12.10, E12.11, E12.2, E12.20, E12.21, E12.3, E12.30, E12.31, E12.4, E12.40, E12.41, E12.5, E12.50, E12.51, E12.6, E12.60, E12.61, E12.7, E12.70, E12.71, E12.8, E12.80, E12.81, E12.9, E12.90, E12.91, E13, E13.0, E13.00, E13.01, E13.02, E13.1, E13.10, E13.11, E13.12, E13.13, E13.14, E13.15, E13.16, E13.2, E13.20, E13.21, E13.22, E13.23, E13.29, E13.3, E13.30, E13.31, E13.32, E13.33, E13.34, E13.35, E13.36, E13.39, E13.4, E13.40, E13.41, E13.42, E13.43, E13.49, E13.5, E13.50, E13.51, E13.52, E13.53, E13.59, E13.6, E13.60, E13.61, E13.62, E13.63, E13.64, E13.65, E13.69, E13.7, E13.70, E13.71, E13.72, E13.73, E13.8, E13.80, E13.81, E13.9, E13.90, E13.91, E14, E14.0, E14.00, E14.01, E14.02, E14.1, E14.10, E14.11, E14.12, E14.13, E14.14, E14.15, E14.16, E14.2, E14.20, E14.21, E14.22, E14.23, E14.29, E14.3, E14.30, E14.31, E14.32, E14.33, E14.34, E14.35, E14.36, E14.39, E14.4, E14.40, E14.41, E14.42, E14.43, E14.49, E14.5, E14.50, E14.51, E14.52, E14.53, E14.59, E14.6, E14.60, E14.61, E14.62, E14.63, E14.64,

	E14.65, E14.69, E14.7, E14.70, E14.71, E14.72, E14.73, E14.8, E14.80, E14.81, E14.9, E14.90, E14.91
Emphysema	J41, J41.0, J41.1, J41.8, J42, J43, J43.0, J43.1, J43.2, J43.8, J43.9, J44, J44.0, J44.1, J44.8, J44.9, J45, J45.0, J45.1, J45.8, J45.9, J46, J47, U83.1, U83.2, U83.3, J98.2, J98.3
Endocarditis	I33, I33.0, I33.9, I38, I39, I39.0, I39.1, I39.2, I39.3, I39.4, I39.8
Heart failure	I11, I11.0, I13.0, I13.1, I13.2, I42, I43, I42, I42.0, I42.1, I42.2, I42.3, I42.4, I42.5, I42.6, I42.7, I42.8, I42.9, I43, I43.0, I43.1, I43.2, I43.8, I50, I50.0, I50.9, I50.1, I13.53, I14.53, I25.5 #NB: does not include U82.2, E10.53, E11.53
Hemiplegia	G80.02, G80.1, G80.2, G81, G81.0, G81.1, G81.9, G82, G82.0, G82.00, G82.01,G82.02, G82.03, G82.04, G82.05, G82.06, G82.1, G82.10, G82.11, G82.12,G82.13, G82.14, G82.15, G82.16, G82.2, G82.20, G82.21, G82.22, G82.23,G82.24, G82.25, G82.26, G82.3, G82.30, G82.31, G82.32, G82.33, G82.34,G82.35, G82.36, G82.4, G82.40, G82.41, G82.42, G82.43, G82.44, G82.45,G82.46, G82.5, G82.51, G82.52, G82.53, G82.54, G82.55, G82.56,U80.5, G11.4, M62.3, M62.30, M62.31, M62.32, M62.33, M62.34, M62.35, M62.36, M62.37, M62.38, M62.39
HIV	B20, B21, B22, B23, B23.0, B23.8, B24, F02.4, O98.7, R75, Z21
Hypertension	I10, I11, I11.0, I11.9, I12, I12.0, I12.9, I13, I13.0, I13.1, I13.2, I13.9, I15, I15.0, I15.1, I15.2, I15.8, I15.9, U82.3
Hyperlipidaemia	E78, E78.0, E78.1, E78.2, E78.3, E78.4, E78.5, E78.6, E78.8, E78.9
Inflammatory bowel disease	K50, K50.0, K50.1, K50.8, K50.9, K51, K51.0, K51.2, K51.3, K51.4,K51.5, K51.8, K51.9, M07.4, M07.40, M07.41, M07.42, M07.43, M07.44,M07.45, M07.46, M07.47, M07.48, M07.49, M07.5, M07.50, M07.51,M07.52, M07.53, M07.54, M07.55, M07.56, M07.57, M07.58, M07.59,M07.6, M07.61, M07.62, M07.63, M07,64, M07.65, M07.66, M07.67,M07.68, M07.69, U84.1, U84.2, M09.1, M09.10, M09.11, M09.12,M09.13, M09.14, M09.15, M09.16, M09.17, M09.18, M09.19, M09.2,M09.20, M09.21, M09.22, M09.23, M09.24, M09.25, M09.26, M09.27,M09.28, M09.29
Ischaemic heart disease	I20, I20.0, I20.1, I20.8, I20.9, I21, I21.0, I21.1, I21.2, I21.3, I21.4, I21.9, I22, I22.0, I22.1, I22.8, I22.9, I23, I23.0, I23.1, I23.2, I23.3, I23.4, I23.5, I23.6, I23.8, I24, I24.0, I24.1, I24.8, I24.9, I25, I25.0, I25.1, I25.10, I25.11, I25.12, I25.13, I25.2, I25.3, I25.4, I25.5, I25.6, I25.8, I25.9
Influenza	J09, J10, J10.0, J10.1, J10.8, J11, J11.0, J11.1, J11.8
Leukaemia	C90.1, C90.10, C90.11, C91, C91.0, C91.00, C91.01, C91.1, C91.10, C91.11, C91.2, C91.20, C91.21, C91.3, C91.30, C91.31, C91.4, C91.40, C91.41, C91.5, C91.50, C91.51, C91.6, C91.60, C91.61, C91.7, C91.70, C91.71, C91.8, C91.80, C91.81, C91.9, C91.90, C91.91, C92, C92.0, C92.00, C92.01, C92.1, C92.10, C92.11, C92.2, C92.20, C92.21, C92.4, C92.40, C92.41, C92.5, C92.50, C92.51, C92.6, C92.60, C92.61, C92.7, C92.70, C92.71, C92.8, C92.80, C92.81, C92.9, C92.90, C92.91, C93, C93.0, C93.00, C93.01, C93.1, C93.10, C93.11, C93.2, C93.20, C93.21, C93.3, C93.30, C93.31, C93.7, C93.70, C93.71, C93.9, C93.90, C93.91, C94, C94.0, C94.00, C94.01, C94.2, C94.20, C94.21, C94.3, C94.30, C94.31, C94.7, C94.70, C94.71, C95, C95.0, C95.00, C95.01, C95.1, C95.10, C95.11,

	C95.2, C95.20, C95.21, C95.7, C95.70, C95.71, C95.9, C95.90, C95.91, D47.5, Z85.6, M9733/3, M9742/3, M980, M9800/3, M9801/3, M9802/3, M9803/3, M9804/3, M9805/3, M9806/3, M9807/3, M9808/3, M9809/3, M981-M983, M9811/3, M9812/3, M9813/3, M9814/3, M9815/3, M9816/3, M9817/3, M9818/3, M9820/3, M9821/3, M9822/3, M9823/3, M9824/3, M9825/3, M9826/3, M9827/3, M982-M983, M9830/3, M9831/1, M9832/3, M9833/3, M9834/3, M9835/3, M9836/3, M9837/3, M9840/3, M984-M993, M9850/3, M9860/3, M9861/3, M9862/3, M9863/3, M9864/3, M9865/3, M9866/3, M9867/3, M9868/3, M9869/3, M9870/3, M9871/3, M9872/3, M9873/3, M9874/3, M9875/3, M9876/3, M9880/3, M9890/3, M9891/3, M9892/3, M9893/3, M9894/3, M9895/3, M9896/3, M9897/3, M9898/3, M9900/3, M9910/3, M9911/3, M994, M9940/3, M9945/3, M9946/3, M9948/3, M9963/3, M9964/3
Liver disease	K70, K70.0, K70.1, K70.2, K70.3, K70.4, K70.9, K71, K71.0, K71.1, K71.2, K71.3, K71.4, K71.5, K71.6, K71.7, K71.8, K71.9, K72, K72.0, K72.1, K72.9, K73, K73.0, K73.1, K73.2, K73.8, K73.9, K74, K74.0, K74.1, K74.2, K74.3, K74.4, K74.5, K74.6, K75,K75.0, K75.1, K75.2, K75.3, K75.4, K75.8, K75.9, K76, K76.0, K76.1, K76.2,K76.3, K76.4, K76.5, K76.6, K76.7, K76.8, K76.9,B18, B18.0, B18.1, B18.2, B18.8, B18.9, Z94.4,I85, I85.0, I85.9, I86.4, I98.2, I98.20, I98.21, I98.3
Moderate to severe liver disease	K70.4, K71.1, K72, K72.0, K72.1, K72.9, K76.7, I85, I85.0, I85.9, I86.4, I98.2, I98.20, I98.21, I98.3
Lymphoma	C81, C81.0, C81.1, C81.2, C81.3, C81.4, C81.7, C81.9, C82, C82.0, C82.1, C82.2, C82.3, C82.4, C82.5, C82.6, C82.7, C82.9, C83, C83.0, C83.1, C83.2, C83.3, C83.4, C83.5, C83.6, C83.7, C83.8, C83.9, C84, C84.2, C84.3, C84.4, C84.5, C84.6, C84.7, C84.8, C84.9, C85, C85.1, C85.2, C85.7, C85.9, C86, C86.0, C86.1, C86.2, C86.3, C86.4, C86.5, C88.4, C88.40, C88.41, C91.5, C91.50, C91.51, C96.3, M959, M9590/3, M9591/3, M9595/3, M9596/3, M9597/3, M959-M972, M9650/3, M9651/3, M9653/3, M9654/3, M9655/3, M9659/3, M965-M966, M9663/3, M9664/3, M9665/3, M9667/3, M9670/3, M9671/3, M9672/3, M9673/3, M9674/3, M9675/3, M9676/3, M9677/3, M9678/3, M9679/3, M967-M969, M967-M972, M9680/3, M9681/3, M9682/3, M9683/3, M9684/3, M9685/3, M9686/3, M9687/3, M9688/3, M9689/3, M969, M9690/3, M9691/3, M9692/3, M9693/3, M9694/3, M9695/3, M9696/3, M9697/3, M9698/3, M9699/3, M9702/3, M9703/3, M9704/3, M9705/3, M9706/3, M9707/3, M9708/3, M9709/3, M970-M971, M971, M9711/3, M9712/3, M9713/3, M9714/3, M9716/3, M9717/3, M9719/3, M972, M9723/3, M9725/3, M9726/3, M9727/3, M9728/3, M9729/3, M9735/3, M9737/3, M9738/3, C88.4, C88.40, C88.41
Myocarditis	I01.2, I09.0, I40, I40.0, I40.1, I40.8, I40.9, I41, I41.0, I41.1, I41.2, I41.8, I51.4
Myositis	M33, M33.0, M33.1, M33.2, M33.9, M60, M60.0, M60.00, M60.01, M60.02, M60.03, M60.04, M60.05, M60.07, M60.07, M60.08, M60.09, M60.1, M60.10, M60.11, M60.12, M60.13, M60.14, M60.15, M60.16, M60.17, M60.18, M60.19, M60.8, M60.80, M60.81, M60.82, M60.83, M60.84, M60.85, M60.86, M60.87,

	M60.88, M60.89, M60.9, M60.90, M60.91, M60.92, M60.93, M60.94, M60.95, M60.96, M06.97, M60.98, M60.99, M63, M63.0, M63.00, M63.01, M63.02, M63.03, M63.04, M63.05, M63.07, M63.07, M63.08, M63.09, M63.1,M63.10, M63.11, M63.12, M63.13, M63.14, M63.15, M63.16, M63.17, M63.18, M63.19, M63.2, M63.20, M63.21, M63.22, M63.23, M63.24, M63.25, M63.26, M63.27, M63.28, M63.29, M63.3, M63.30, M63.31, M63.32, M63.33, M63.34, M63.35, M63.36, M63.37, M63.38, M63.39
Pericarditis	I01.0, I09.2, I30, I30.1, I30.8, I30.9, I31.0, I31.1, I32, I32.0, I32.1, I32.8
Psoriatic arthritis	M07.0, M07.00, M07.04, M07.07, M07.09, M07.1, M07.10, M07.12, M07.13, M07.14, M07.15, M07.16, M07.17, M07.18, M07.19, M07.2, M07.3, M07.30, M07.31, M07.32, M07.33, M07.34, M07.35, M0.36, M07.37, M07.38, M07.39
Peripheral vascular disease	W09.01, E09.02, E09.5, E09.51, E09.52, E10.51, E10.52, E11.51,E11.52, E13.51, E13.52, E14.51, E14.52, I70, I70.0, I70.1, I70.2,I70.20, I70.21, I70.22, I70.23, I70.24, I70.8, I70.9, I71, I71.0,I71.00, I71.01, I71.02, I71.03, I71.1, I71.2, I71.3, I71.4, I71.5,I71.6, I71.8, I71.9, I72, I72.0 I72.1, I72.2, I72.3, I72.4, I72.5,I72.6, I72.8, I72.9, I73, I73.0, I73.1, I73.8, I73.9, I74, I74.0,I74.1, I74.2, I74.3, I74.4, I74.5, I74.8, I74.9, I77, I77.0, I77.1,I77.2, I77.3, I77.4, I77.5, I77.6, I77.8, I77.9, I78, I78.0, I78.1,I78.8, I78.9, I79, I79.0, I79.1, I79.2, I79.8
Rheumatoid arthritis	M05, M05.0, M05.00, M05.01, M05.02, M05.03, M05.04, M05.05, M05.06, M05.07, M05.08, M05.09, M05.1, M05.10, M05.11, M05.12, M05.13, M05.14, M05.15, M05.16, M05.17, M05.18, M05.19, M05.2, M05.20, M05.21, M05.22, M05.23, M05.24, M05.25, M05.26, M05.27, M05.28, M05.29, M05.3, M05.30, M05.31, M05.32, M05.33, M05.34, M05.35, M05.36, M05.37, M05.38, M05.39, M05.8, M05.80, M05.81, M05.82, M05.83, M05.84, M05.85, M05.86, M05.87, M05.88, M05.89, M05.9, M05.90, M05.91, M05.92, M05.93, M05.94, M05.95, M05.96, M05.97, M05.98, M05.99, M06, M06.0, M06.00, M06.01, M06.02, M06.03, M06.04, M06.05, M06.06, M06.07, M06.08, M06.09, M06.2, M06.20, M06.21, M06.22, M06.23, M06.24, M06.25, M06.26, M06.27, M06.28, M06.29, M06.3, M06.30, M06.31, M06.32, M06.33, M06.34, M06.35, M06.36, M06.37, M06.38, M06.39, M06.8, M06.80, M06.81, M06.82, M06.83, M06.84, M06.85, M06.86, M06.87, M06.88, M06.89, M06.9, M06.90, M06.91, M06.92, M06.93, M06.94, M06.95, M06.96, M06.97, M06.98, M06.99, U86.1
Sarcoidosis	D86, D86.0, D86.1, D86.2, D86.3, D86.8, D86.9, G53.2, M63.3, M63.30, M63.31, M63.32, M63.33, M63.34, M63.35, M63.36, M63.37, M63.38, M63.39))#NB: there is no code for cardiac sarcoidosis of any form
Systemic lupus erythematosus	L93, L93.0, L93.1, L93.2, M32, M32.0, M32.1, M32.8, M32.9, U86.3
Smoking	Z58.7, F17, F17.0, F17.2, F17.3, F17.4, F17.6, F17.7, F17.8, F17.9, T65.2, Z71.6, Z72.0, Z86.43

Stroke	G46, G46.0, G46.1, G46.2, G46.3, G46.4, G46.5, G46.6, G46.7, G46.8, I63, I63.0, I63.1, I63.2, I63.3, I63.4, I63.5, I63.6, I63.8, I63.9, I69.3, I60, I60.0, I60.1, I60.2, I60.3, I60.4, I60.5, I60.6, I60.7, I60.8, I60.9, I61, I61.0, I61.1, I61.2, I61.3, I61.4, I61.5, I61.6, I61.8, I61.9, I62, I62.0, I62.1, I62.9, I64, I69.0, I69.1, I69.2, I64, I69.4
Syncope	R55
Transient ischaemic attack	G45, G45.0, G45.1, G45.2, G45.3, G45.4, G45.8, G45.9
Ventricular arrhythmia	I47.0, I47.2, I49.0
Venous thromboembolism	I26, I26.0, I26.9, I80, I80.0, I80.1, I80.2, I80.3, I80.8, I80.9, I81, I82, I82.0, I82.1, I82.2, I82.3, I82.8, I82.9
Autoimmune disease	D86, D86.0, D86.1, D86.2, D86.3, D86.8, D86.9, G53.2, L93, L93.0, L93.1, L93.2, MO6, MO6, MO7, MO8, MO9, M30, M31, M32, M33, M34, M35, M36, M45, M46, M63.3, M63.30, M63.31, M63.32, M63.33, M63.34, M63.35, M63.36, M63.37, M63.38, M63.39, U86.3
Malignancy	ICD-10 codes beginning with C
Respiratory disease	ICD-10 codes beginning with J
Digestive disease	ICD-10 codes beginning with K
Chest MRI	90901-04
Defibrillator insertion	38393-00
Heart transplantation	90205-00, 90205-01
Hip fracture	47528-01, 49318-00, 49319-00
Myocardial biopsy	38275-00, 38418-03

Table S2: Comparative trends for myocarditis based on sex

Feature	Male	Female	p-value
In hospital mortality	112/2690 (4.2%)	70/1381 (5.1%)	0.214
ICU admission	388/2690 (14.4%)	237/1381 (17.2%)	0.025
Mechanical ventilation	185/2690 (6.9%)	130/1381 (9.4%)	0.005
Autoimmune myocarditis	90/2690 (3.3%)	139/1381 (10.1%)	<0.001
Malignant myocarditis	178/2690 (6.6%)	143/1381 (10.4%)	<0.001
Reactive myocarditis	939/2690 (34.9%)	598/1381 (43.3%)	<0.001

Continuous variables reported as median (IQR). Binary variables reported as mean (n).
Where denominators were less than 4,071 this indicated missing data.

Table S3: Comparative trends for myocarditis in major cities and rural areas

Feature	Major city	Rural area	p-value
ICU admission	481/3007 (16.0%)	31/235 (13.2%)	0.297
Mechanical ventilation	248/3007 (8.2)	13/235 (5.5%)	0.177
In hospital mortality	129/3007 (4.3%)	12/235 (5.1%)	0.671
Biopsy during or after diagnosis	181/3007 (6.0%)	8/235 (3.4%)	0.133
Charlson comorbidity index	1 (0-3)	1 (0-3)	0.062

a. Major city defined by Australia Statistical Geography Standard of 1

b. Rural area defined by Australian Statistical Geography Standard of 3 or more

Continuous variables reported as median (IQR). Binary variables reported as mean (n).

Where denominators were less than 4,071 this indicated missing data.

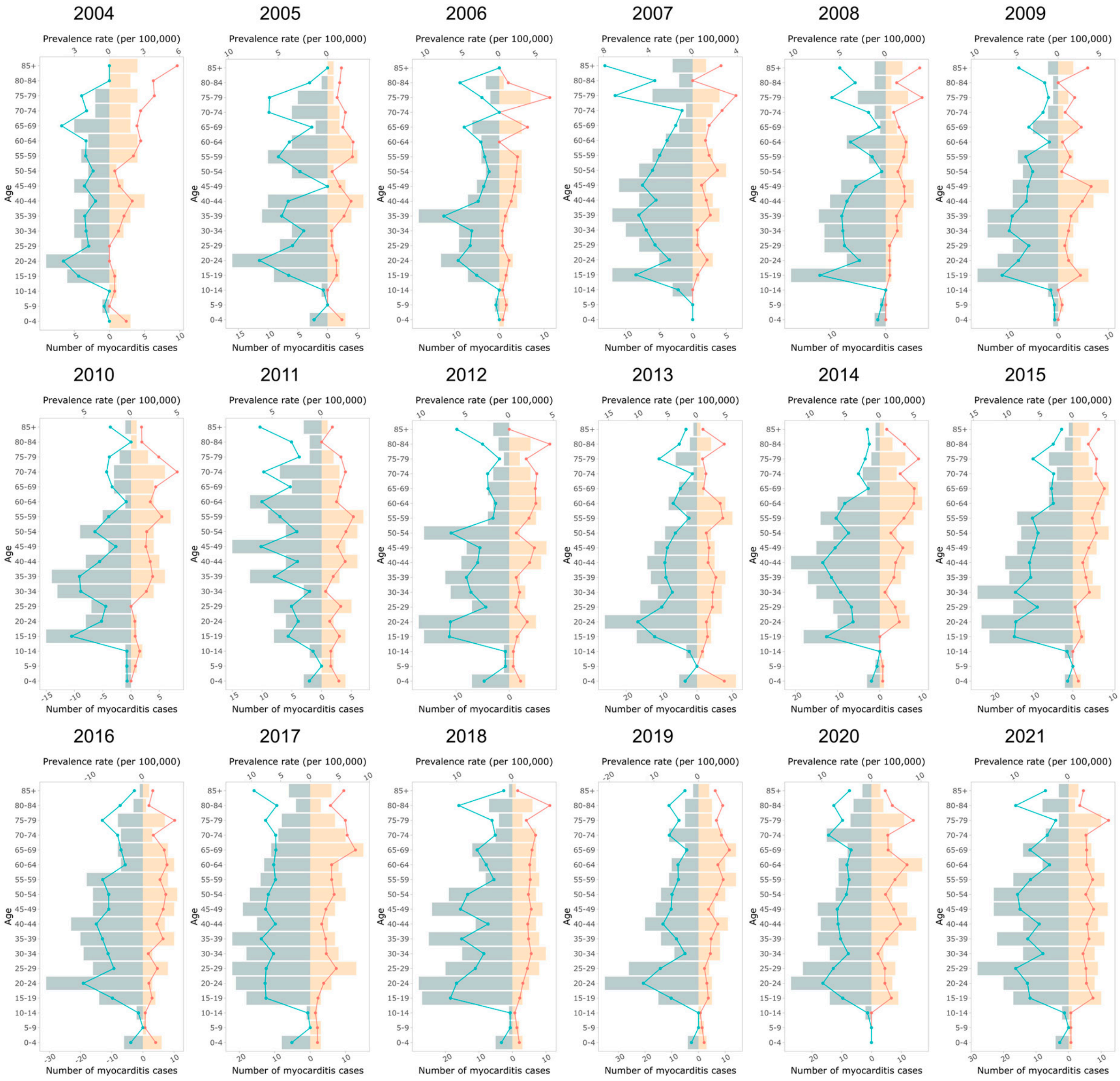

Figure S1: Population demographics of first presentation of myocarditis per year separated by age and sex

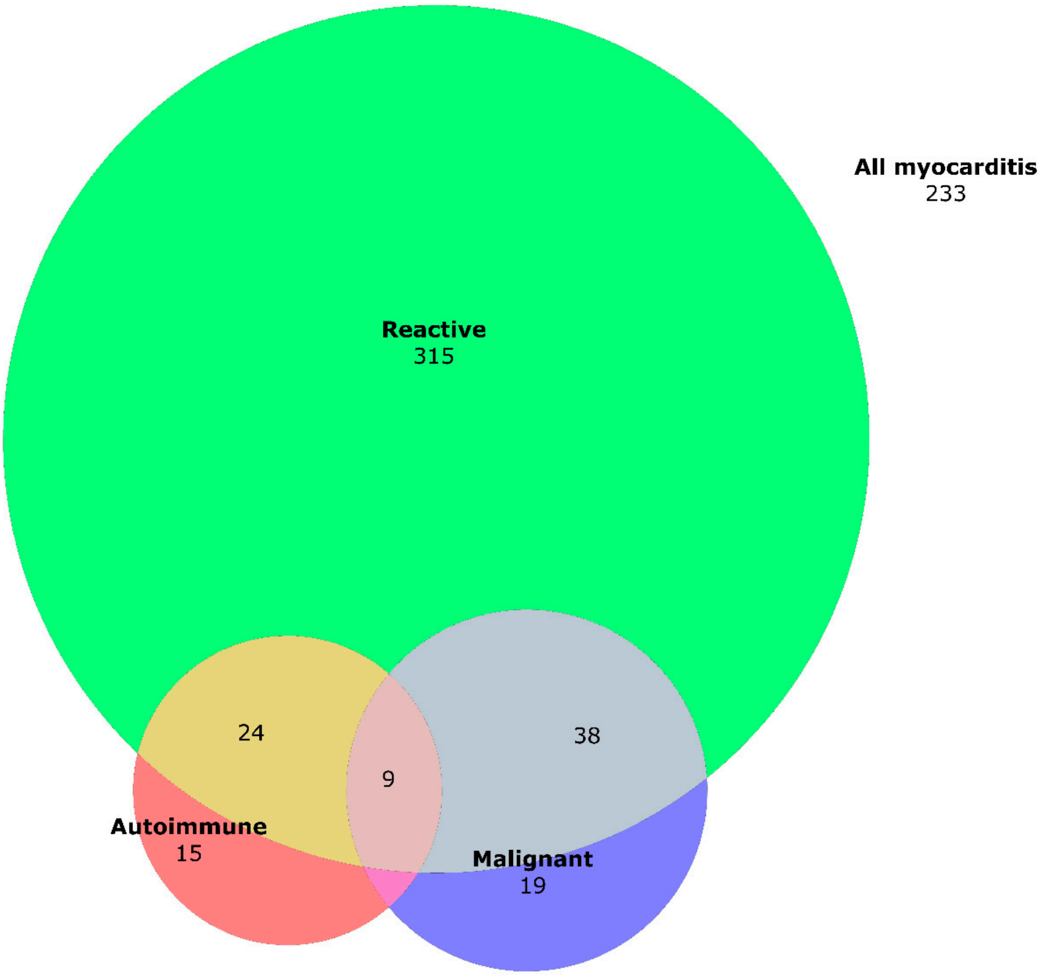

Figure S2: Frequency of myocarditis according to possible classification since 25 January 2020. Autoimmune, malignant aetiologies were defined by having a background of autoimmune disease, or respectively. Reactive myocarditis was defined as having a history of a respiratory or digestive system infection in the past 30 days. Covid-19 associated myocarditis accounted for 281 cases.

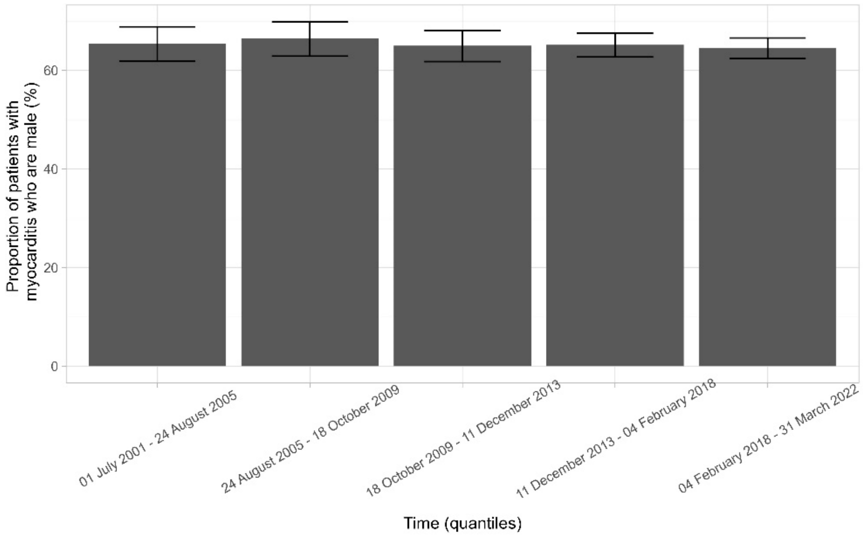

Proportion of patients with myocarditis who are male (%)
0
20
40
60
01 July 2001 - 24 August 2005
24 August 2005 - 18 October 2009
18 October 2009 - 11 December 2013
11 December 2013 - 04 February 2018
04 February 2018 - 31 March 2022
Time (quantiles)

(a)

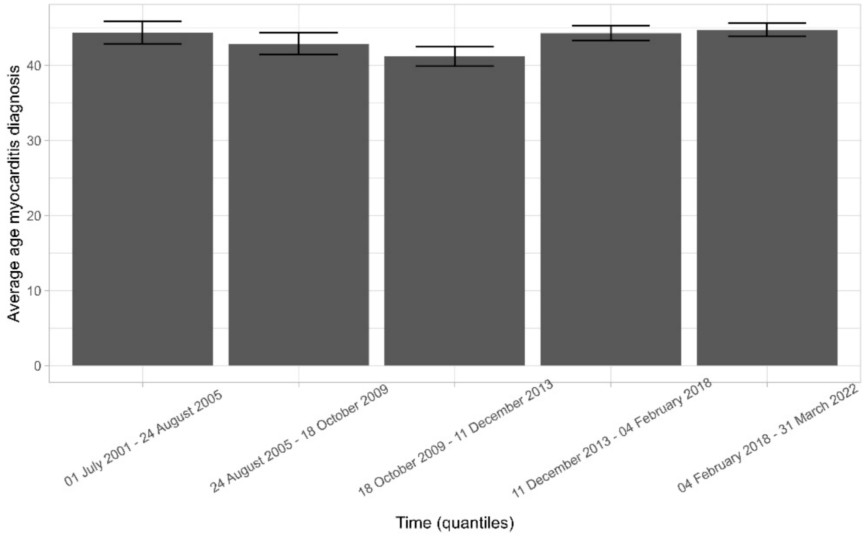

Average age myocarditis diagnosis
0
10
20
30
40
01 July 2001 - 24 August 2005
24 August 2005 - 18 October 2009
18 October 2009 - 11 December 2013
11 December 2013 - 04 February 2018
04 February 2018 - 31 March 2022
Time (quantiles)

(b)

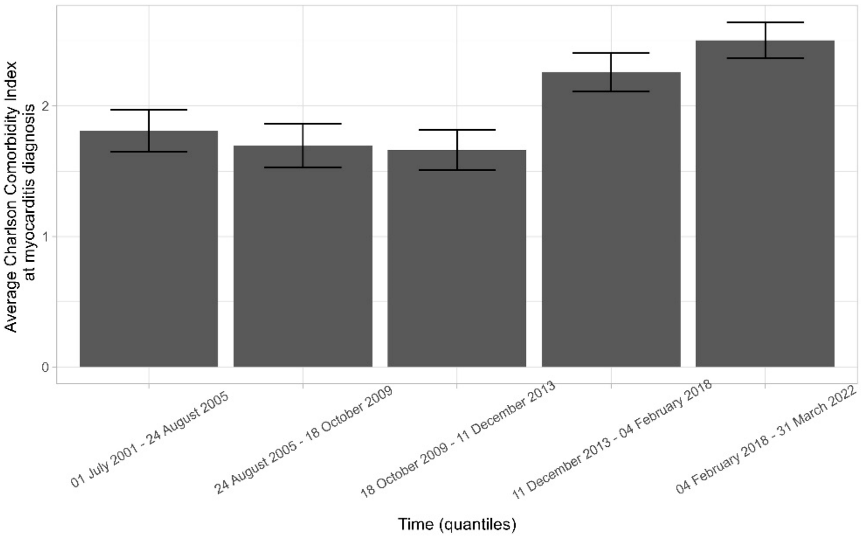

Average Charlson Comorbidity Index at myocarditis diagnosis
0
1
2
01 July 2001 - 24 August 2005
24 August 2005 - 18 October 2009
18 October 2009 - 11 December 2013
11 December 2013 - 04 February 2018
04 February 2018 - 31 March 2022
Time (quantiles)

(c)

(d)

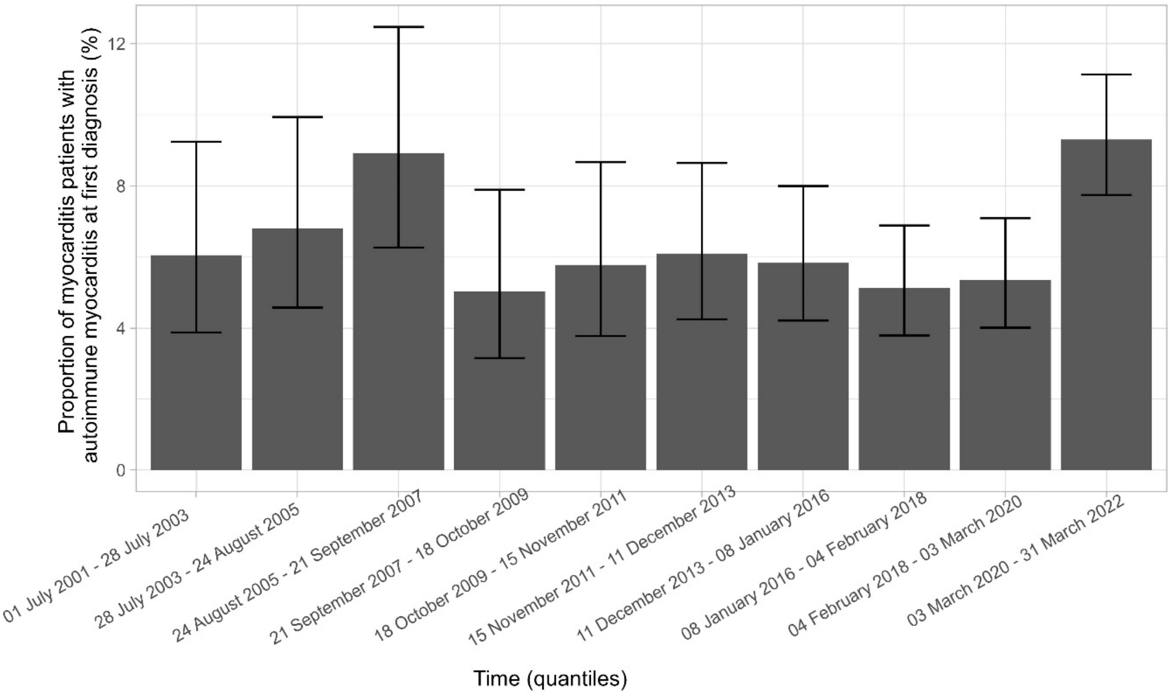

Proportion of myocarditis patients with autoimmune myocarditis at first diagnosis (%)
12
8
4
0
01 July 2001 - 28 July 2003
28 July 2003 - 24 August 2005
24 August 2005 - 21 September 2007
21 September 2007 - 18 October 2009
18 October 2009 - 15 November 2011
15 November 2011 - 11 December 2013
11 December 2013 - 08 January 2016
08 January 2016 - 04 February 2018
04 February 2018 - 03 March 2020
03 March 2020 - 31 March 2022
Time (quantiles)

(e)

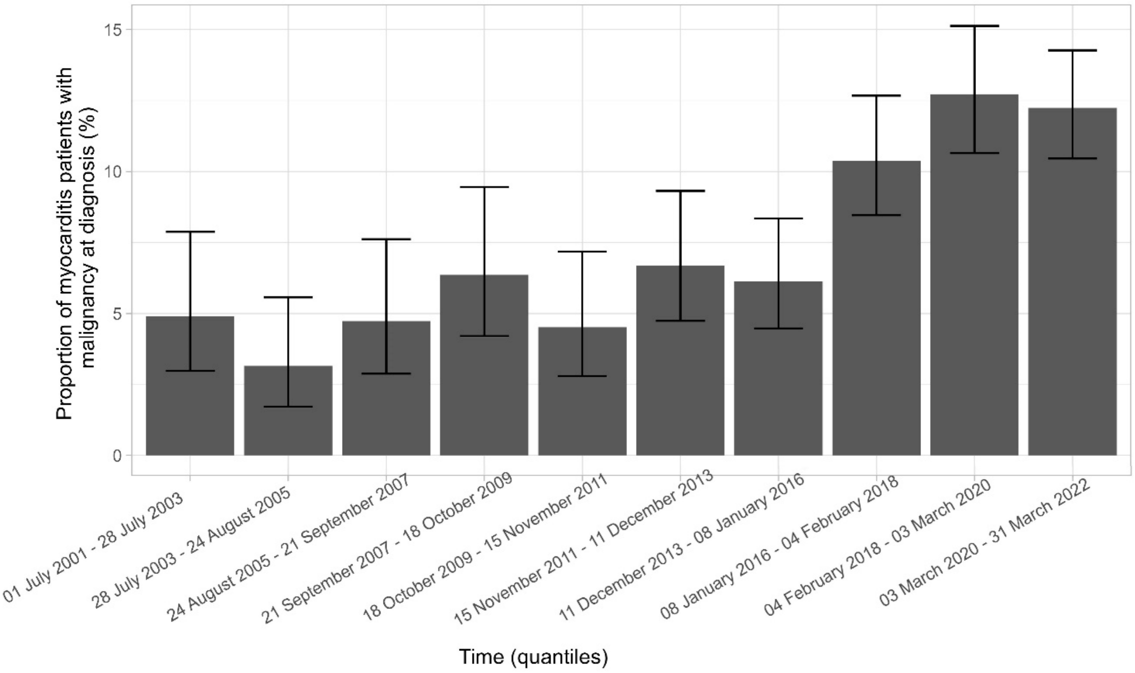

Proportion of myocarditis patients with malignancy at diagnosis (%)
15
10
5
0
01 July 2001 - 28 July 2003
28 July 2003 - 24 August 2005
24 August 2005 - 21 September 2007
21 September 2007 - 18 October 2009
18 October 2009 - 15 November 2011
15 November 2011 - 11 December 2013
11 December 2013 - 08 January 2016
08 January 2016 - 04 February 2018
04 February 2018 - 03 March 2020
03 March 2020 - 31 March 2022
Time (quantiles)

(f)

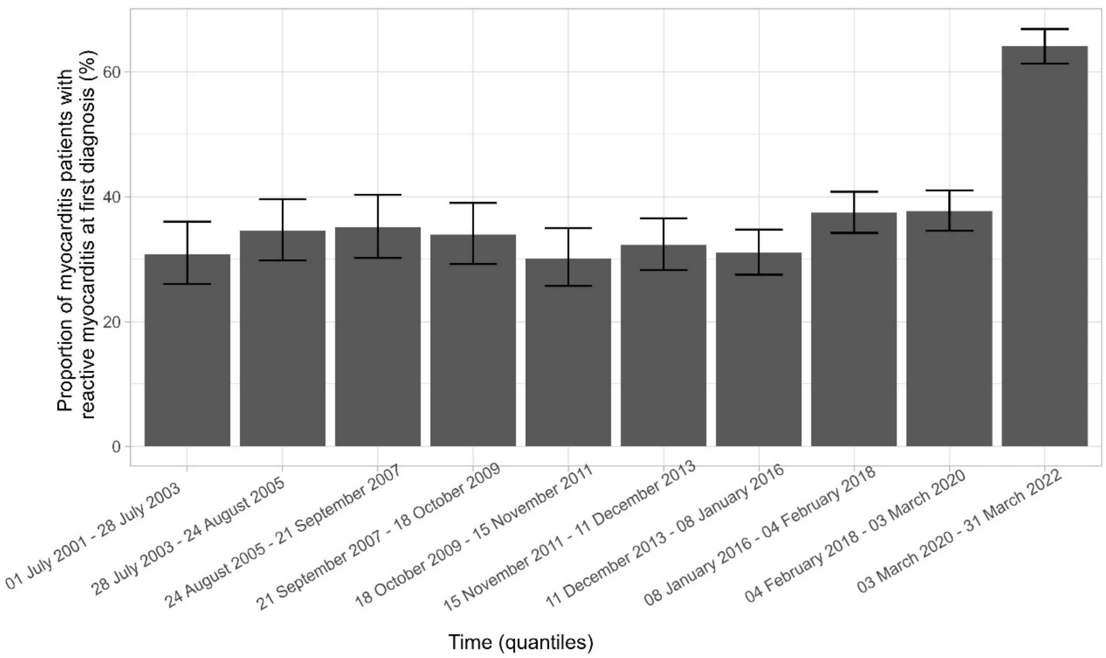
Proportion of myocarditis patients with reactive myocarditis at first diagnosis (%)
0
20
40
60
01 July 2001 - 28 July 2003
28 July 2003 - 24 August 2005
24 August 2005 - 21 September 2007
21 September 2007 - 18 October 2009
18 October 2009 - 15 November 2011
15 November 2011 - 11 December 2013
11 December 2013 - 08 January 2016
08 January 2016 - 04 February 2018
04 February 2018 - 03 March 2020
03 March 2020 - 31 March 2022
Time (quantiles)

(g)

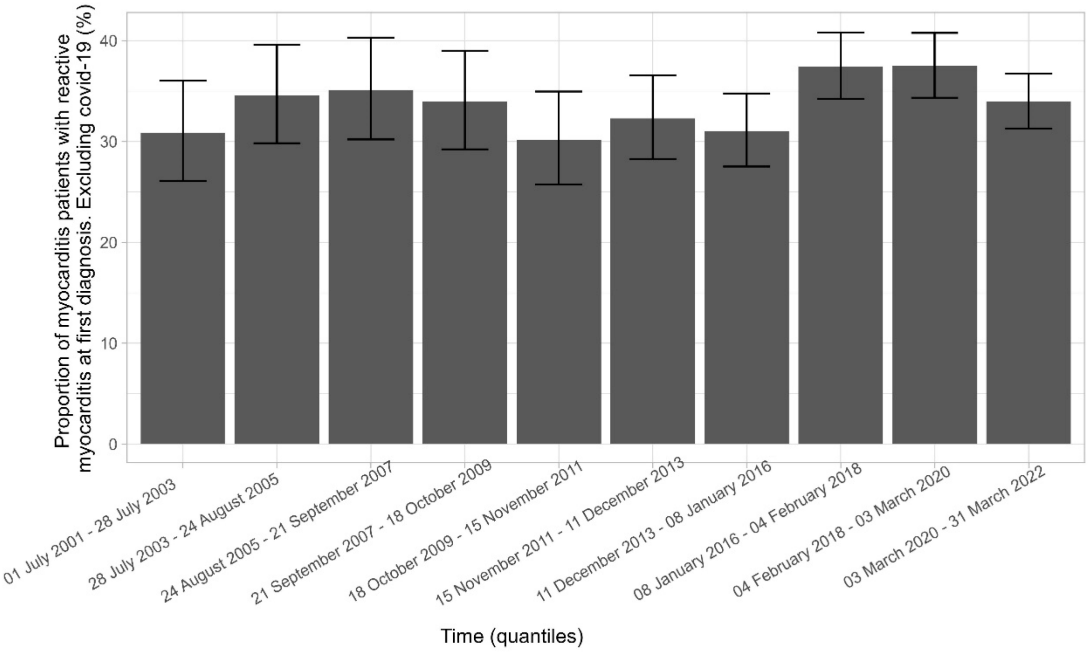
Proportion of myocarditis patients with reactive myocarditis at first diagnosis. Excluding covid-19 (%)
0
10
20
30
40
01 July 2001 - 28 July 2003
28 July 2003 - 24 August 2005
24 August 2005 - 21 September 2007
21 September 2007 - 18 October 2009
18 October 2009 - 15 November 2011
15 November 2011 - 11 December 2013
11 December 2013 - 08 January 2016
08 January 2016 - 04 February 2018
04 February 2018 - 03 March 2020
03 March 2020 - 31 March 2022
Time (quantiles)

(h)

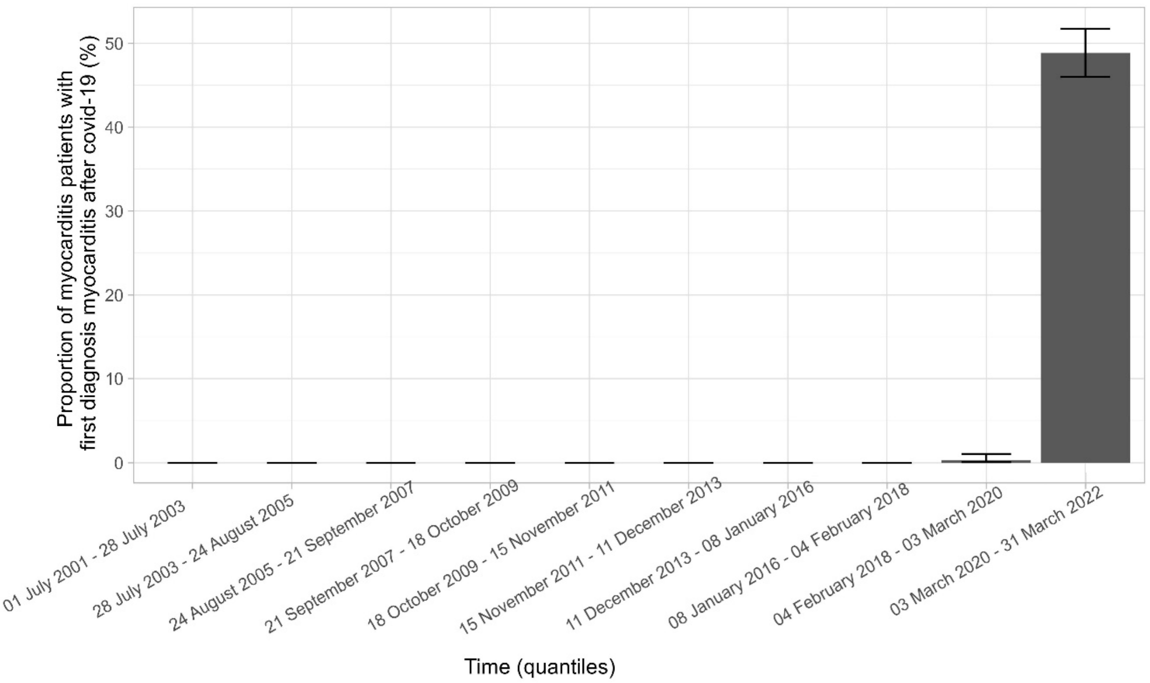
Proportion of myocarditis patients with first diagnosis myocarditis after covid-19 (%)
50
40
30
20
10
0
01 July 2001 - 28 July 2003
28 July 2003 - 24 August 2005
24 August 2005 - 21 September 2007
21 September 2007 - 18 October 2009
18 October 2009 - 15 November 2011
15 November 2011 - 11 December 2013
11 December 2013 - 08 January 2016
08 January 2016 - 04 February 2018
04 February 2018 - 03 March 2020
03 March 2020 - 31 March 2022
Time (quantiles)

(i)

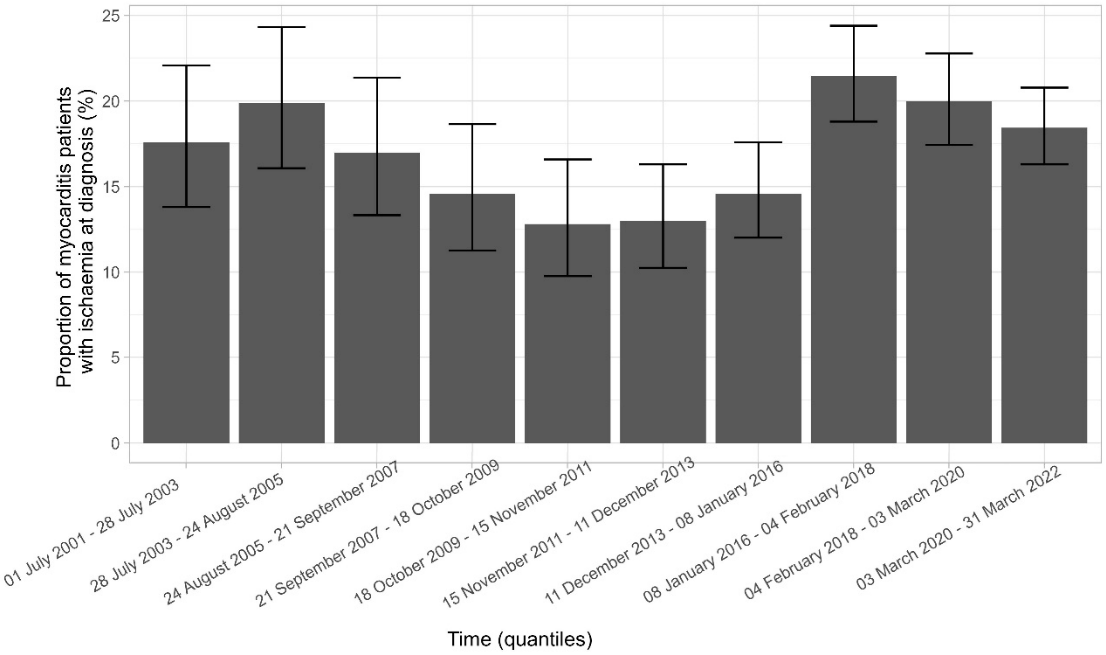
Proportion of myocarditis patients with ischaemia at diagnosis (%)
25
20
15
10
5
0
01 July 2001 - 28 July 2003
28 July 2003 - 24 August 2005
24 August 2005 - 21 September 2007
21 September 2007 - 18 October 2009
18 October 2009 - 15 November 2011
15 November 2011 - 11 December 2013
11 December 2013 - 08 January 2016
08 January 2016 - 04 February 2018
04 February 2018 - 03 March 2020
03 March 2020 - 31 March 2022
Time (quantiles)

(j)

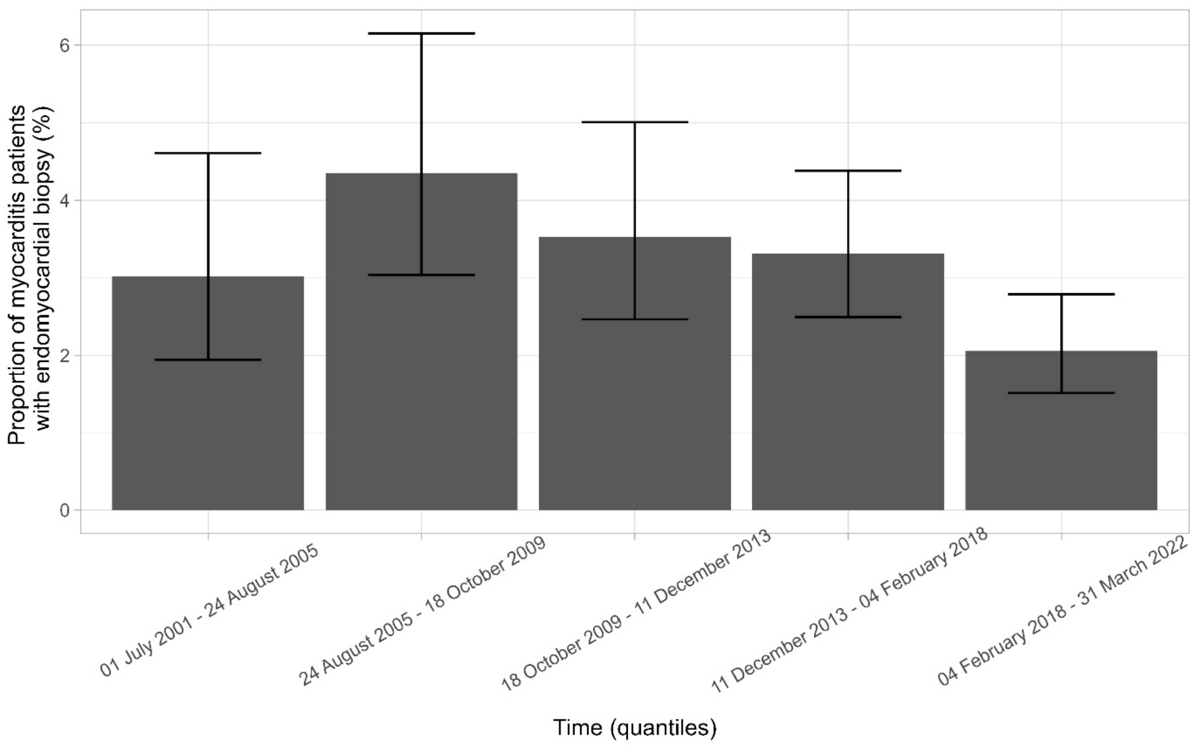
Proportion of myocarditis patients with endomyocardial biopsy (%)
0
2
4
6
01 July 2001 - 24 August 2005
24 August 2005 - 18 October 2009
18 October 2009 - 11 December 2013
11 December 2013 - 04 February 2018
04 February 2018 - 31 March 2022
Time (quantiles)

(k)

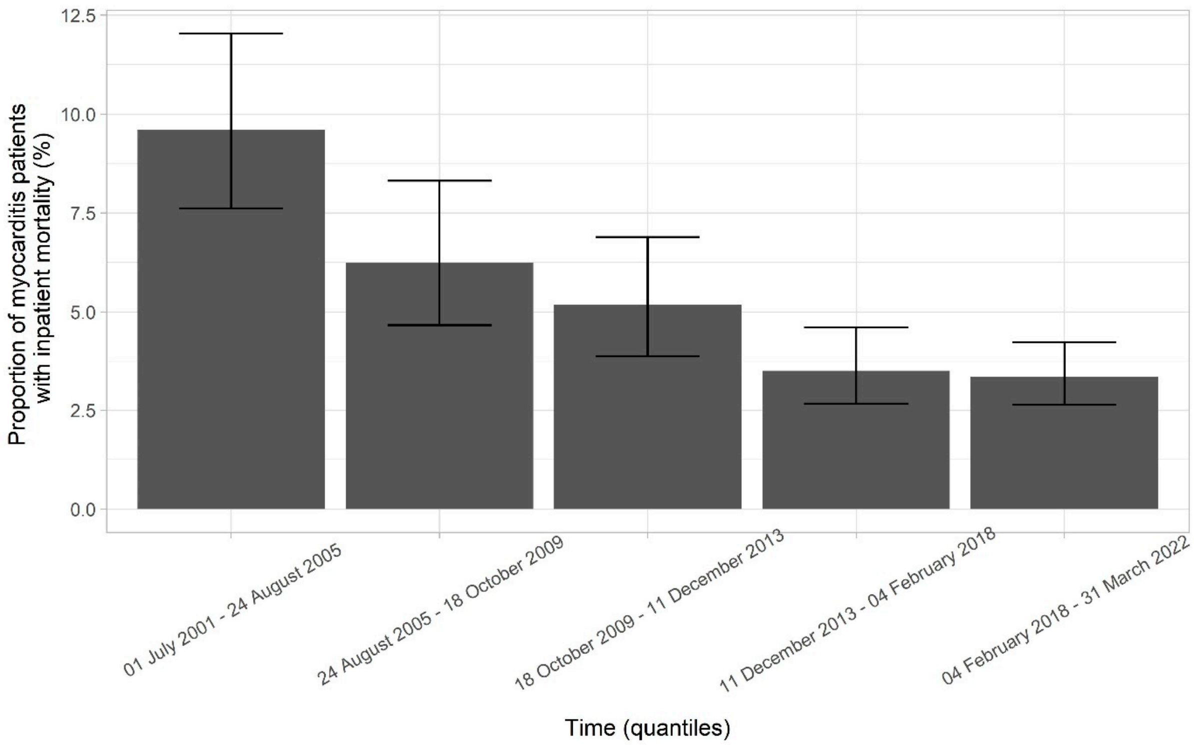
Proportion of myocarditis patients with inpatient mortality (%)
0.0
2.5
5.0
7.5
10.0
12.5
01 July 2001 - 24 August 2005
24 August 2005 - 18 October 2009
18 October 2009 - 11 December 2013
11 December 2013 - 04 February 2018
04 February 2018 - 31 March 2022
Time (quantiles)

(l)

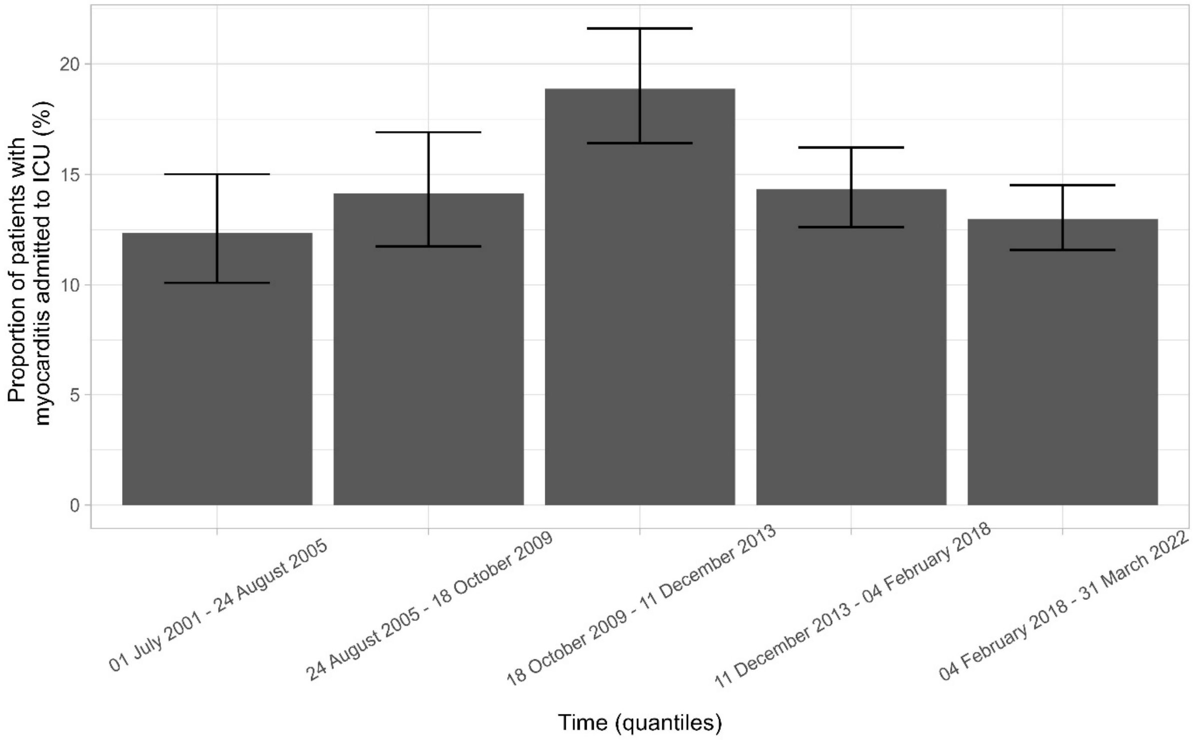
Proportion of patients with myocarditis admitted to ICU (%)
0
5
10
15
20
01 July 2001 - 24 August 2005
24 August 2005 - 18 October 2009
18 October 2009 - 11 December 2013
11 December 2013 - 04 February 2018
04 February 2018 - 31 March 2022
Time (quantiles)

(m)

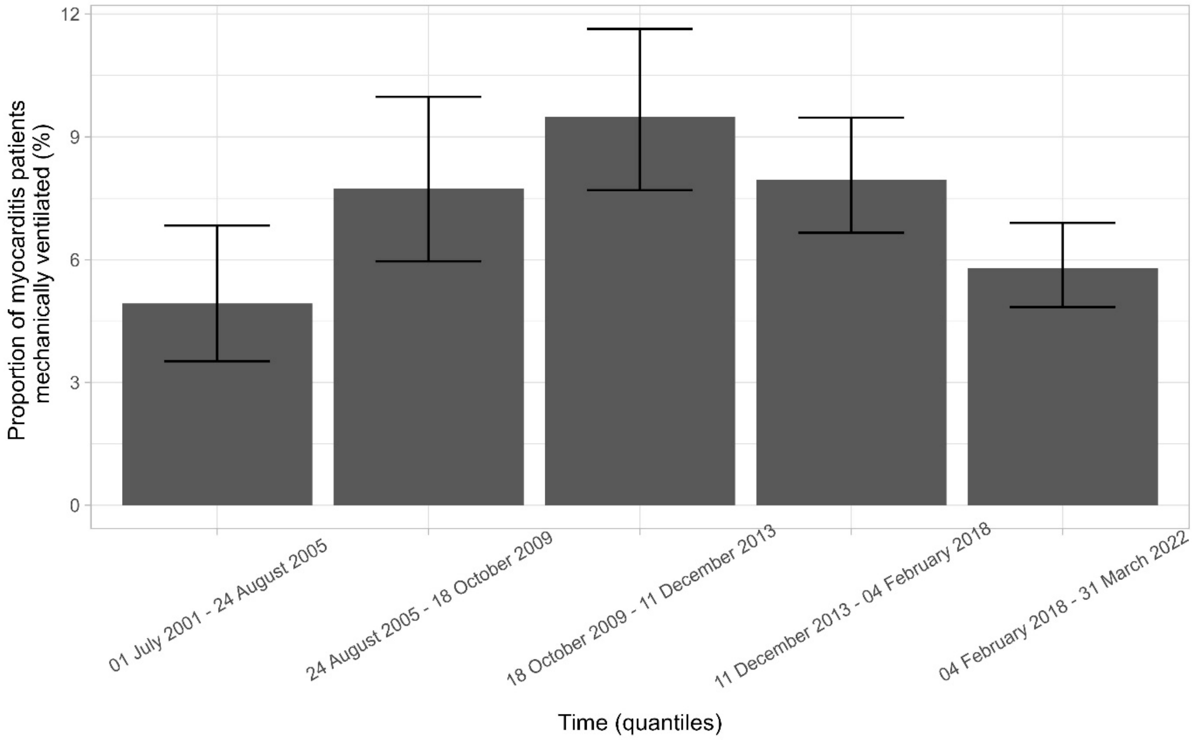
Proportion of myocarditis patients mechanically ventilated (%)
0
3
6
9
12
01 July 2001 - 24 August 2005
24 August 2005 - 18 October 2009
18 October 2009 - 11 December 2013
11 December 2013 - 04 February 2018
04 February 2018 - 31 March 2022
Time (quantiles)

(n)

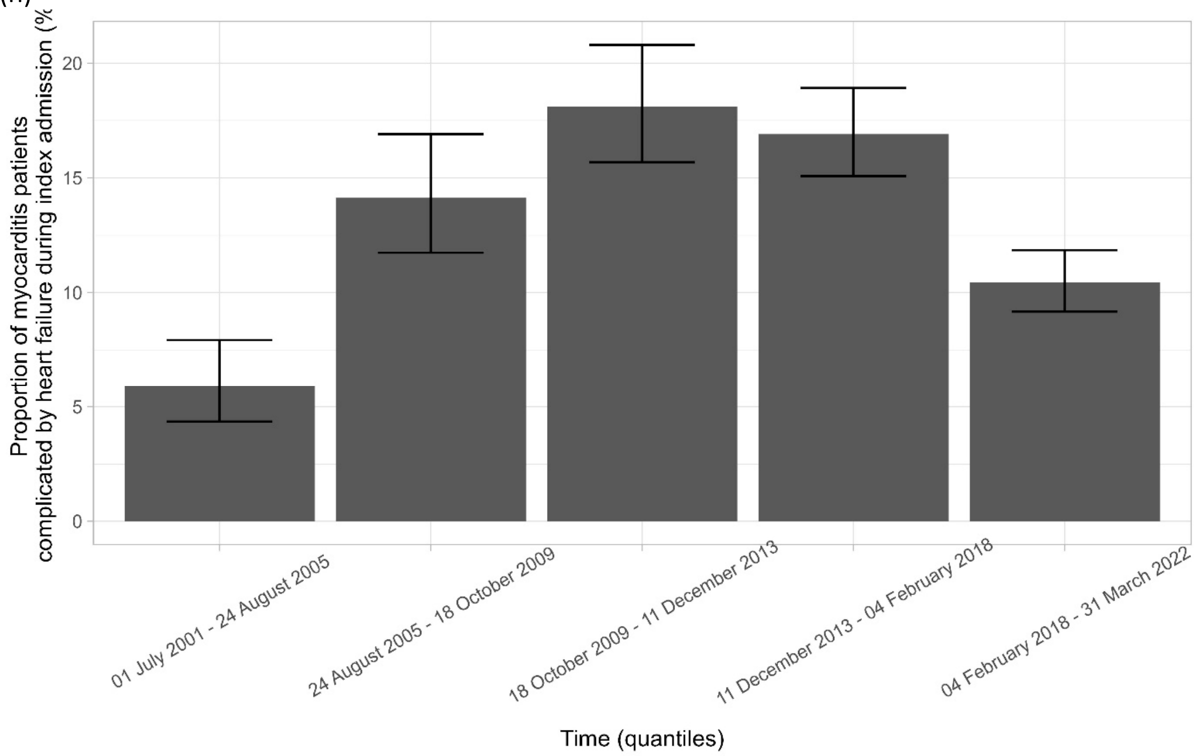
Proportion of myocarditis patients
complicated by heart failure during index admission (%
20
15
10
5
0
01 July 2001 - 24 August 2005
24 August 2005 - 18 October 2009
18 October 2009 - 11 December 2013
11 December 2013 - 04 February 2018
04 February 2018 - 31 March 2022
Time (quantiles)

(o)

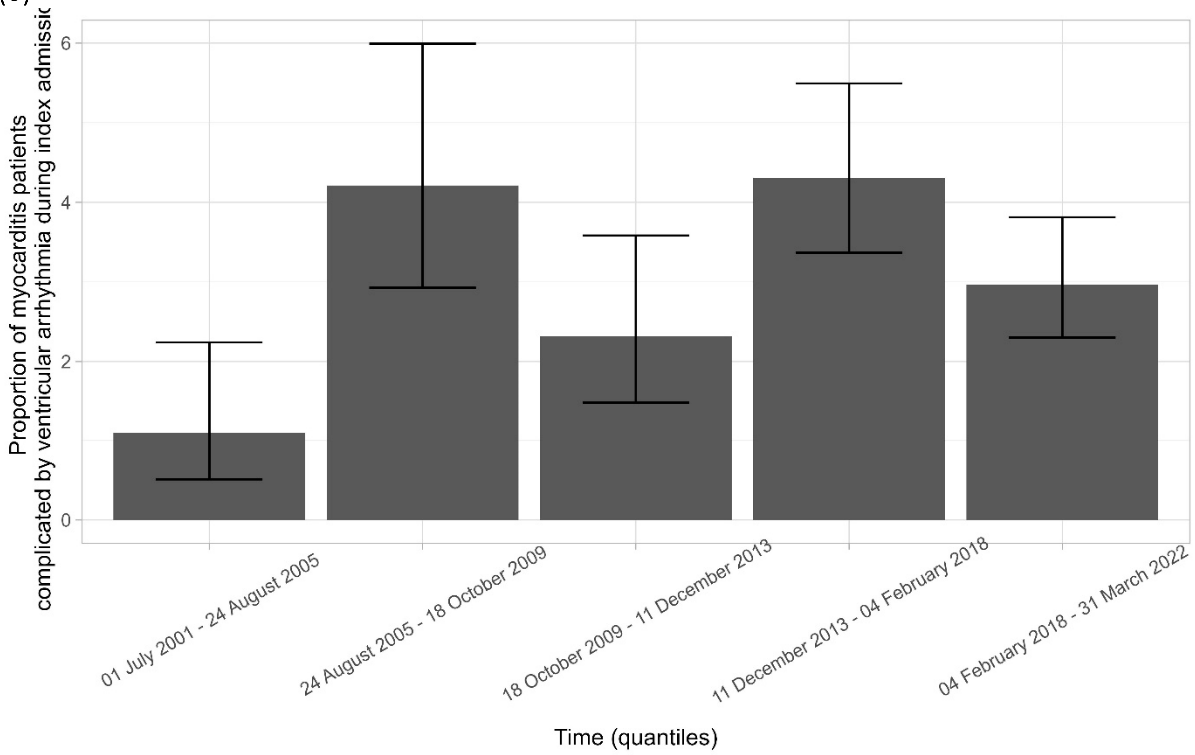
Proportion of myocarditis patients
complicated by ventricular arrhythmia during index admissio
6
4
2
0
01 July 2001 - 24 August 2005
24 August 2005 - 18 October 2009
18 October 2009 - 11 December 2013
11 December 2013 - 04 February 2018
04 February 2018 - 31 March 2022
Time (quantiles)

(p)

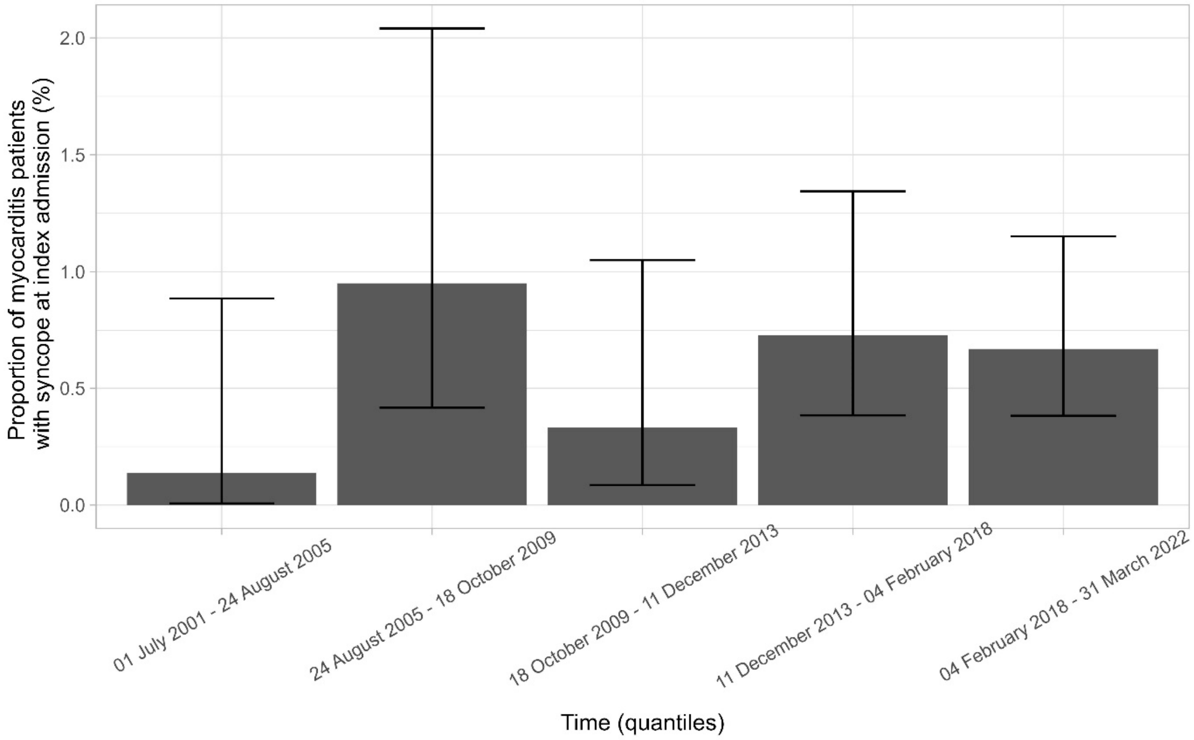
Proportion of myocarditis patients with syncope at index admission (%)
2.0
1.5
1.0
0.5
0.0
01 July 2001 - 24 August 2005
24 August 2005 - 18 October 2009
18 October 2009 - 11 December 2013
11 December 2013 - 04 February 2018
04 February 2018 - 31 March 2022
Time (quantiles)

(q)

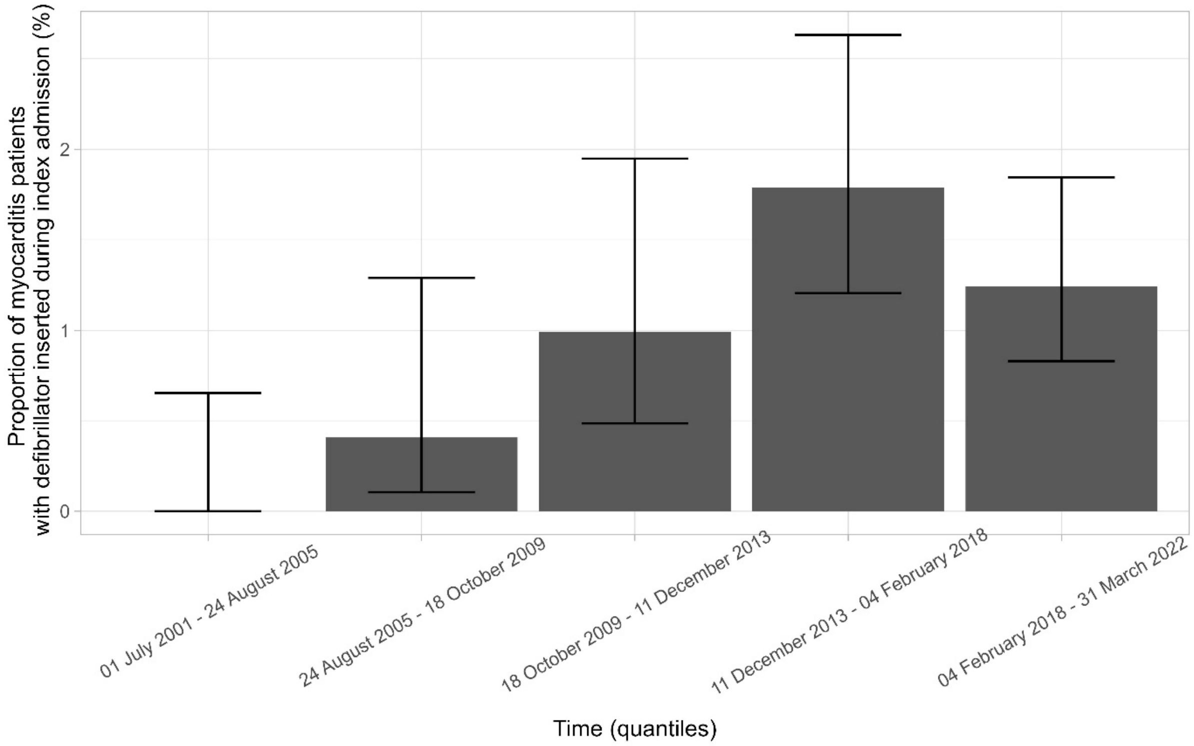
Proportion of myocarditis patients with defibrillator inserted during index admission (%)
2
1
0
01 July 2001 - 24 August 2005
24 August 2005 - 18 October 2009
18 October 2009 - 11 December 2013
11 December 2013 - 04 February 2018
04 February 2018 - 31 March 2022
Time (quantiles)

(r)

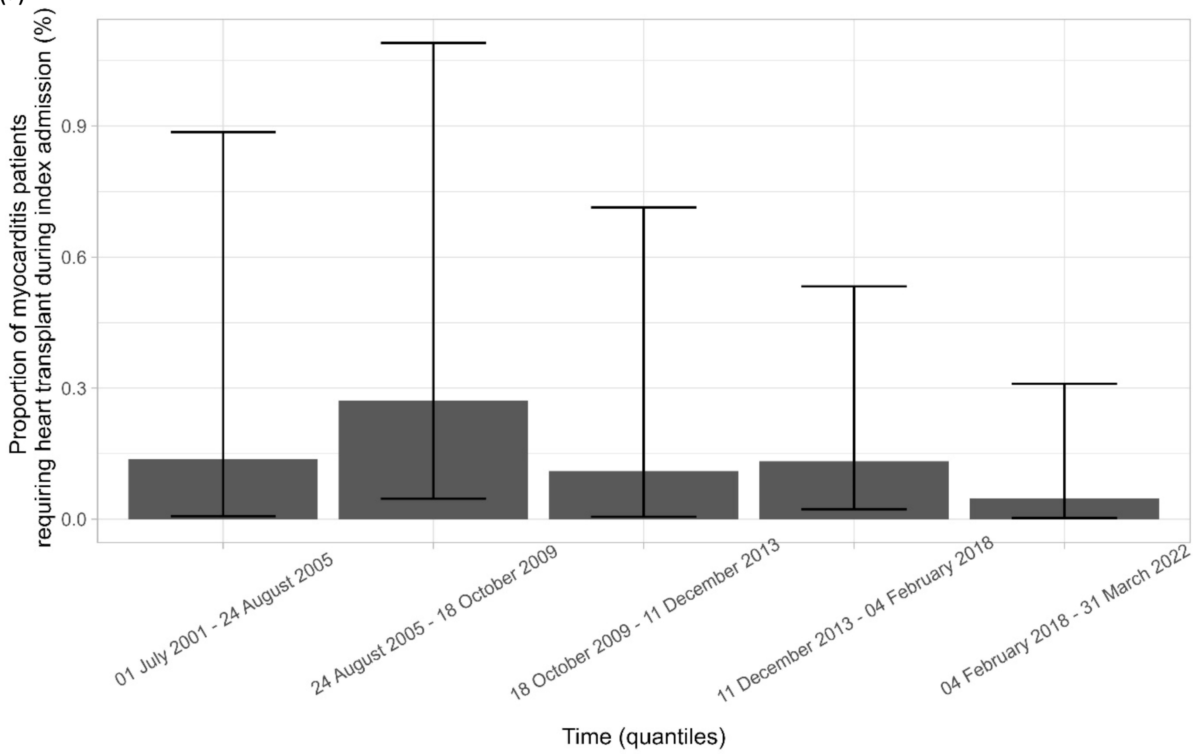

Figure S3: Changing myocarditis demographics over time from 2001 to 2022
(a) sex (p=0.209 by logistic regression)
(b) age (p=0.002 by linear regression)
(c) Charlson comorbidity index (p<0.001 by linear regression)
(d) autoimmune (p=0.563 by logistic regression)
(e) malignant (p<0.001 by logistic regression)
(f) reactive (p<0.001 by logistic regression)
(g) reactive, excluding covid-19 (p=0.227 by logistic regression)
(h) Covid-19 associated
(i) ischaemia (p=0.046 by logistic regression)
(j) biopsy during same admission (p=0.031 by logistic regression)
(k) In hospital mortality (OR 1.11 95% CI 1.08-1.14 reduction in mortality per year, p <0.001 by adjusted logistic regression)
(l) ICU admission (OR 1.02 95% CI 1.01-1.04 reduction in ICU admissions per year, p=0.009 by adjusted logistic regression)
(m) Mechanical ventilation (OR 1.04 95% CI 1.02-1.06 reduction in mechanical ventilation per year, p<0.001 by adjusted logistic regression)
(n) Heart failure during myocarditis admission (OR 1.05 95% CI 1.03-1.06 reduction in heart failure per year, p<0.001 by adjusted logistic regression)
(o) Ventricular arrhythmia during myocarditis admission (OR 1.00, 95% CI 0.97-1.03 reduction in ventricular arrhythmia per year, p=0.925 by adjusted logistic regression)
(p) Syncope during/presentation of myocarditis (OR 0.97 95% CI 0.91-1.04, p=0.463 by adjusted logistic regression)
(q) Defibrillator insertion during myocarditis admission (OR 0.90, 95% CI 0.85-0.95 reduction in defibrillator insertion per year, p<0.001 by adjusted logistic regression)
(r) Heart transplant during myocarditis index admission (OR 1.18, 95% CI 1.01-1.39, p=0.037 by adjusted logistic regression)
Classification determined according to classification of first presentation of myocarditis.

Multivariable adjustment was for age, sex and Charlson Comorbidity Index. Plotted figures are crude numbers.

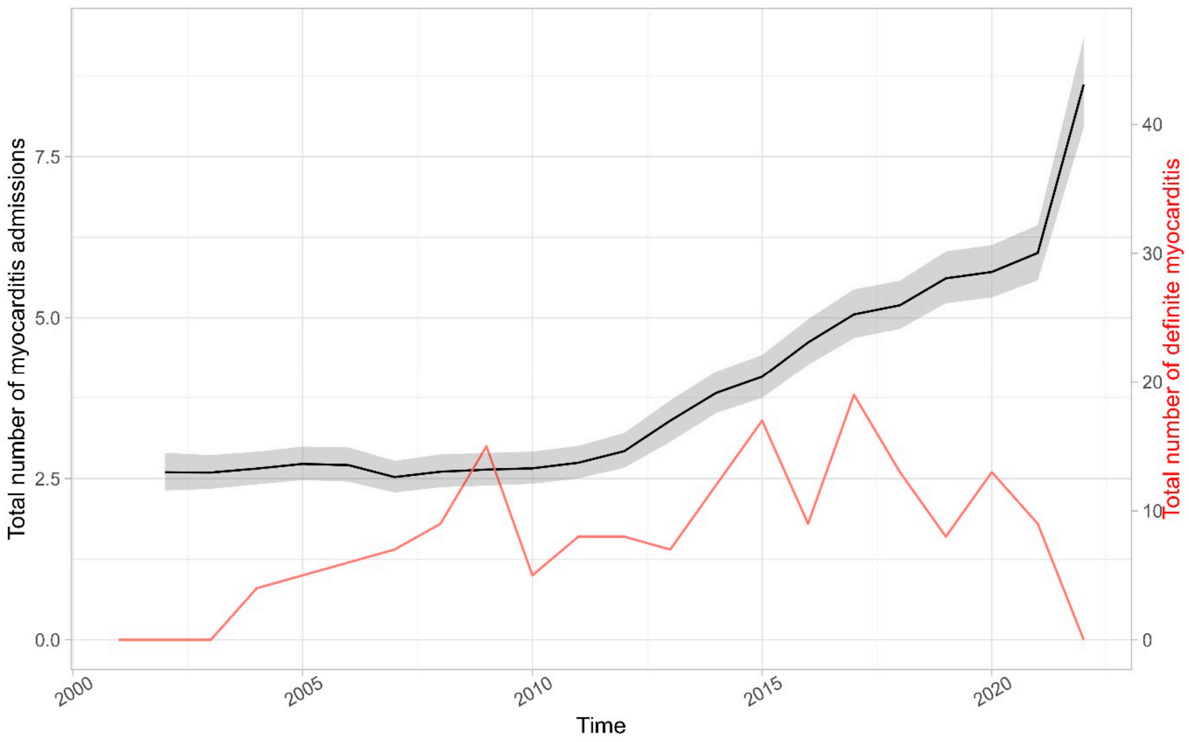

Figure S4: Incidence of definitive myocarditis admissions in NSW over time plotted with total myocarditis admissions

Shaded area indicates 95% credible intervals.
